# Low-frequency activity in the subthalamic nucleus informs about the acute neuropsychiatric state in Parkinson’s disease

**DOI:** 10.1038/s41531-025-01233-3

**Published:** 2026-01-15

**Authors:** Elena Bernasconi, Alberto Averna, Valentina D’Onofrio, Deborah Amstutz, Damian M. Herz, Laura Alva, Andreia D. Magalhães, Katrin Petermann, Ines Debove, M. Lenard Lachenmayer, Andreas Nowacki, Claudio Pollo, Paul Krack, Mario Sousa, Gerd Tinkhauser

**Affiliations:** 1 Department of Neurology, Bern University Hospital and University of Bern, Bern, Switzerland, Bern, Switzerland; 2 Graduate School of Cellular and Biomedical Sciences, University of Bern, Bern, Switzerland; 3 Department of Biomedical Research, University of Bern, Bern, Switzerland; 4 Padova Neuroscience Center, University of Padua, Padua, Italy; 5 Department of Neurology, Heidelberg University Hospital, Heidelberg, Germany; 6 Department of Neurosurgery, Bern University Hospital and University of Bern, Bern, Switzerland

**Keywords:** Diseases, Medical research, Neurology, Neuroscience

## Abstract

Sensing-guided deep brain stimulation (DBS) offers potential to further optimize symptom control in Parkinson’s disease (PD) patients. Emerging evidence suggests that basal ganglia signals reflect not only motor, but also chronic neuropsychiatric symptoms. However, it remains unclear whether local field potentials (LFPs) can inform about acute neuropsychiatric states in PD, which we address in this work. Fourteen PD patients implanted with a brain-sense-enabled neurostimulator underwent an acute levodopa challenge OFF/ON stimulation one year after surgery. In each condition, resting state STN-LFPs were recorded, and the acute neuropsychiatric state was evaluated using the Neuropsychiatric Fluctuation Scale. The relationship between neuropsychiatric state and fluctuation scores with STN low-frequency activity (4–12 Hz) was assessed. An acute low neuropsychiatric state in the OFF-medication condition was associated with elevated theta/low-alpha power. Moreover, the 6–8 Hz activity was indicative of the neuropsychiatric state change following medication intake. Those results were most evident in recordings from the ventral contacts closer to the limbic STN, while chronic stimulation settings covering the dorsal associative and motor STN captured a similar trend. STN low-frequency activity may serve as a biomarker for the acute neuropsychiatric state and neuropsychiatric responsiveness to dopamine and may inform future sensing-guided DBS strategies.

## Introduction

Parkinson’s disease (PD) is characterized by a multifaceted clinical spectrum including both motor and non-motor symptoms^1,2^. Among these, neuropsychiatric symptoms significantly impair patients’ quality of life, and growing efforts are undertaken to better diagnose and treat them^3–5^. Sensing-guided deep brain stimulation (DBS) now enables real-time adjustments of stimulation based on neurophysiological biomarkers^6–9^. However, current biomarker research has predominantly focused on motor symptoms, while biomarkers for frequently occurring neuropsychiatric symptoms remain largely unexplored^10–18^. A few studies show that sub-beta oscillations (4–12 Hz) recorded closer to the ventral limbic subthalamic nucleus (STN) can be related to affective processes^19–21^. Additionally, an association of ventral STN activity with specific chronic neuropsychiatric states has been demonstrated, such as increased alpha power indicating trait impulsivity, decreased alpha power apathy severity, and increased theta power trait anxiety^22–25^. Importantly, while some studies reported stronger immediate local field potential (LFP) responses to emotional stimuli in the right STN^26–28^, others found no lateralization of neuropsychiatric biomarkers^19,22–24,29^. Furthermore, the low-frequency power range is known to increase with dopaminergic medication, while DBS seems not to consistently affect this spectral range^30–33^. Recently introduced sensing technologies now enable the wireless and more flexible recording of subcortical signals in chronically implanted patients, thereby overcoming earlier limitations of short recording windows during DBS surgery or postoperative electrode externalization, where signal quality could be affected by the stun effect^6,34^. This more comfortable and less strenuous setting may be in particular an advantage for investigating neuropsychiatric symptoms biomarkers. Moreover, the translation from biomarkers associated with chronic neuropsychiatric traits to real-time detection of acute neuropsychiatric states has not yet been possible due to the lack of validated instruments capturing momentary neuropsychiatric states. The recently developed Neuropsychiatric Fluctuations Scale (NFS) now allows to sensitively capture and quantify acute neuropsychiatric states and their fluctuations induced by medication and stimulation in PD^35–40^. In contrast to the Movement Disorder Society—Unified Parkinson’s Disease Rating Scale part III (MDS-UPDRS-III), capturing only motor symptoms directly related to the underlying disease, the NFS quantifies the full spectrum of neuropsychiatric symptoms, ranging from hypodopaminergic symptoms (such as low mood, anxiety, or reduced motivation) to hyperdopaminergic symptoms (such as elevated mood, increased creativity, or heightened energy) that can emerge as a consequence of dopaminergic therapy. In this pilot study, we take advantage of this new clinical neuropsychiatric assessment tool, combining it with brain-sense technology to investigate the link between neuronal STN oscillations and acute neuropsychiatric states. The primary objective of this study is not only to advance our pathophysiological understanding of PD, but also to provide the theoretical basis to integrate neuropsychiatric fluctuations in brain-sense guided treatment strategies.

## Results

### Clinical-neurophysiological assessment

**Fig. 1:**
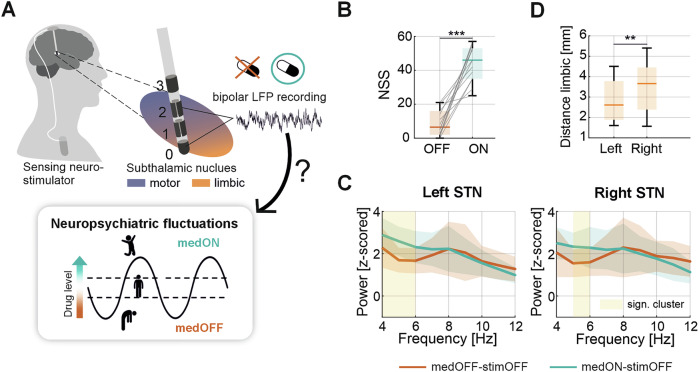
Medication effect on neuropsychiatric state and STN oscillations. **A** Schematic illustration of a multi-contact directional DBS lead implanted in the STN and connected to a sensing-enabled neurostimulator. The relationship between LFPs recorded with the bipolar 0–2 configuration near the limbic STN and the NSS in the respective medication condition was analyzed ON and OFF dopaminergic medication. **B** The boxplot presents the distribution of the NSS of all subjects in the medication OFF (red) and ON (blue) conditions (OFF stimulation). **C** Shows the power spectrum from 4 to 12 Hz averaged across all subjects ±SD in the medication OFF and ON conditions. The shaded area (yellow) indicates a significant difference in power. **D** Illustrates the Euclidean distance from the averaged bipolar sensing configuration (0–2) to the center of the limbic STN in the left (median = 2.61, ranging from 1.60 to 4.50) and right (median = 3.66, ranging from 1.57 to 5.40) hemisphere. STN subthalamic nucleus, LFP local field potentials, DBS deep brain stimulation, NSS neuropsychiatric state score, SD standard deviation.

The relationship between the spectral properties of the LFPs recorded at rest and the neuropsychiatric state OFF and ON dopaminergic medication was analyzed. The full power spectrum of all four conditions can be found in the Supplementary Fig. 1. We first report the results for the conditions when stimulation was switched OFF and LFPs recorded bilaterally with the most ventral bipolar sensing configuration (contact level 0–2) closest to the limbic STN (Fig. 1A). The patient’s acute neuropsychiatric state measured using the Neuropsychiatric State Score (NSS) improved from the OFF (NSS 9.1 ± 7.7 points, mean ± standard deviation (SD)) to the ON medication state (NSS 43.5 ± 10.5 points) by 35.4 ± 15.3 points (*n* = 14, *p* < 0.001) (Fig. 1B), which is in line with previous reports^37–39^. The motor performance improved in all patients upon medication intake, with a decrease of 21.0 ± 9.2 points (47 ± 15%) in the MDS-UPDRS-III (*n* = 14, *p* < 0.001) (Table 1 and Supplementary Fig. 2). Note, the motor state was not significantly correlated with the NSS in the respective conditions. In contrast, the score of the Questionnaire for Impulsive-Compulsive Disorders in Parkinson’s Disease—Rating Scale (QUIP-RS) was significantly positively correlated with the NSS ON medication (rho = 0.61, *p* = 0.028) and the NSS delta between medication ON and OFF condition (rho = 0.63, *p* = 0.022), while the Hospital Anxiety and Depression Scale (HADS) score for anxiety was significantly negatively correlated with the NSS OFF medication (rho = −0.76, *p* = 0.003). The Starkstein Apathy Scale and the HADS for depression showed no significant correlation with the NSS nor NSS delta (Supplementary Fig. 3). The medication dose administered to the patients was on average 230 mg (ranging from 150 to 300 mg) of liquid formulation of levodopa, showing no significant correlation with neither the NSS nor the MDS-UPDRS-III scores (Supplementary Fig. 3). In the ON medication condition, LFP power significantly increased in the theta range in the left (1 Hz resolution; cluster from 4 to 6 Hz, *p* = 0.002) and right hemisphere (cluster from 5 to 6 Hz, *p* = 0.039) (Fig. 1C), while no significant differences were found in the direct comparison of the power spectrum between both hemispheres (Supplementary Fig. 4). Note, the average bipolar 0–2 sensing contact location in the left hemisphere was slightly closer to the limbic center of the STN compared to the right hemisphere (*W* = 11, *p* = 0.007) (Fig. 1D).

**Table 1:** Demographic data

Subject	Age (y)	Disease duration (y)	Months after surgery	Chronic total LEDD	Chronic agonist LEDD only	Levodopa dose [mg]	MDS UPDRS-III med OFF/ON	MoCA	Stimulation level (contacts)	Parameters A/PW/Fs [mA]/[µs]/[Hz]	Chronic 1: same conf. 0: diff. conf
01	59	11	12	740	160	250	34/18	27	Le: C + L3(4, 5, 6)- Ri: C + L3(12, 13, 14)-	Le: 3/60/125 Ri: 3.2/60/125	1
02	59	14	16	580	80	200	51/21	27	Le: C + L3(4, 5, 6)- Ri: C + L3(12, 13, 14)-	Le: 2.4/60/125 Ri: 2.4/60/125	1
03	63	17	12	510	110	200	59/21	29	Le: C + L3(4, 5, 6)- Ri: C + L3(12, 13, 14)-	Le: 2.4/60/125 Ri: 1.8/60/125	1
04	49	9	12	530	0	200	43/21	25	Le: C + L3(4, 5, 6)- Ri: C + L3(12, 13, 14)-	Le: 3.3/60/125 Ri: 2.0/60/125	1
05	59	17	12	900	110	300	55/30	29	Le: C + L3(4, 5, 6)- Ri: C + L3(12, 13, 14)-	Le: 1.6/60/125 Ri: 2.7/60/125	1
06	64	8	12	300	0	200	50/35	28	Le: C + L3(4,5,6)- Ri: C + L3(12,13,14)-	Le: 1.7/60/125 Ri: 1.8/60/125	1
07	58	9	12	580	20	150	36/15	27	Le: C + L3(4, 5, 6)- Ri: C + L3(12, 13, 14)-	Le: 2.4/60/125 Ri: 2.1/60/125	1
08	70	10	11	150	150	200	43/30	22	Le: C + L3(4, 5, 6)- Ri: C + L2.5(9, 10, 11, 12, 13, 14)-	Le: 2.0/60/125 Ri: 1.9/60/125	0
09	71	8	12	980	180	300	42/21	22	Le: C + L3(4, 5, 6)- Ri: C + L3(12, 13, 14)-	Le: 2.3/60/125 Ri: 2.5/60/125	1
10	64	11	12	540	240	300	33/15	28	Le: C + L3(4, 5, 6)- Ri: C + L3(12, 13, 14)-	Le: 1.7/60/125 Ri: 1.7/60/125	1
11	68	11	12	350	150	150	42/33	24	Le: C + L3(4, 5, 6)- Ri: C + L3(12, 13, 14)-	Le: 2.1/60/125 Ri: 1.9/60/125	1
12	39	7	11	590	210	300	54/15	30	Le: C + L2.5(1, 2, 3, 4, 5, 6)- Ri: C + L2.5(9, 10, 11, 12, 13, 14)-	Le: 1.4/60/125 Ri: 1.4/60/125	0
13	74	25	12	950	300	300	47/33	26	Le: C + L3(4, 5, 6)- Ri: C + L3(12, 13, 14)-	Le: 2.6/60/125 Ri: 2.7/60/125	1
14	69	6	12	840	80	200	29/16	27	Le: C + L3(4, 5, 6)- Ri: C + L3(12, 13, 14)-	Le: 2.7/60/125 Ri: 1.9/60/125	1

*MDS-UPDRS-III* Movement Disorder Society—Unified Parkinson’s disease rating scale part III, *y* years, *LEDD* levodopa equivalent daily dose, *MoCA* Montreal Cognitive Assessment, *C* case, *L* level, *A* amplitude, *PW* pulse width, *Fs* stimulation frequency.

### Neurophysiological correlate of the neuropsychiatric state

**Fig. 2:**
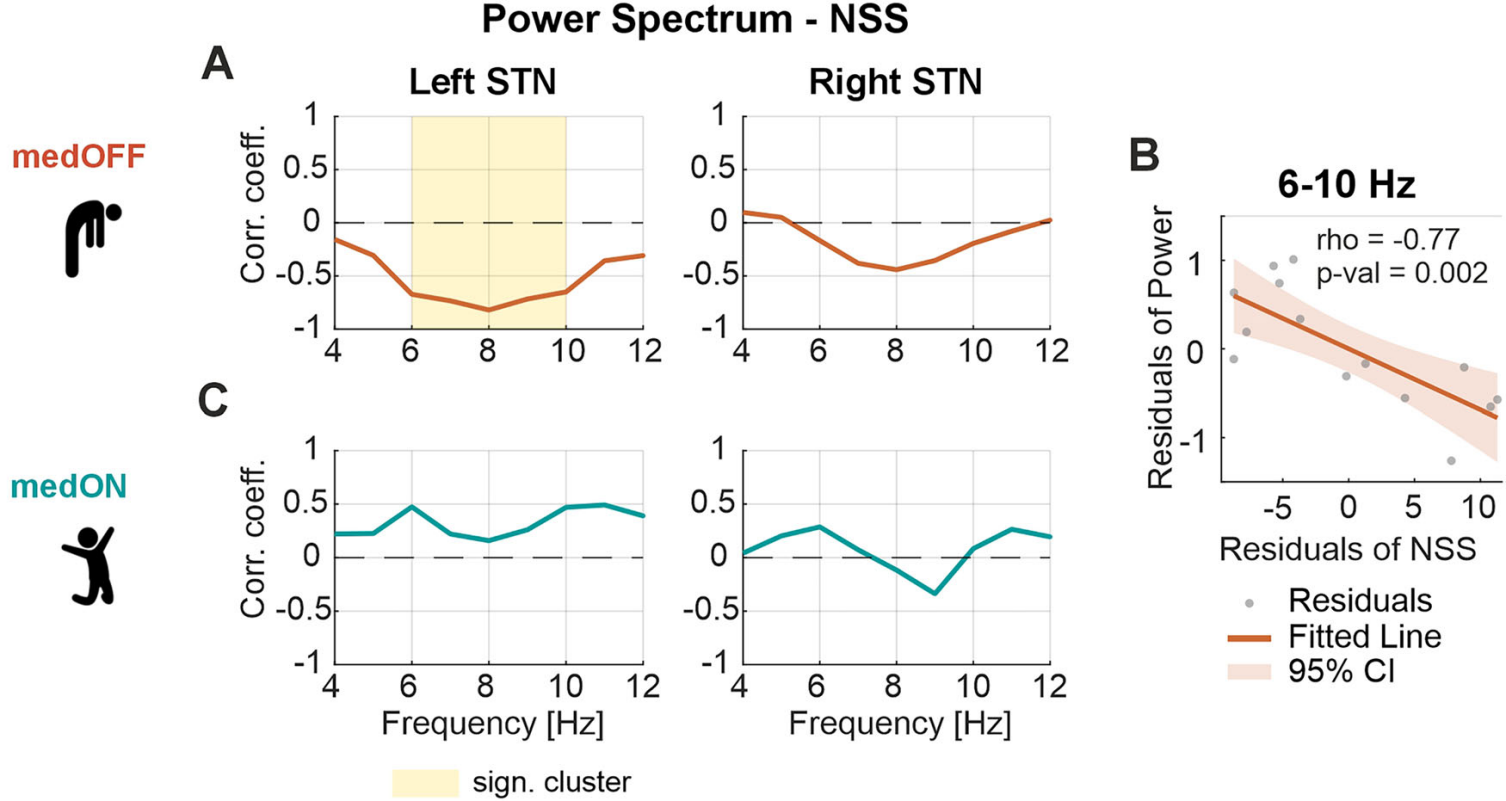
Neurophysiological correlate of the acute neuropsychiatric state. **A** Illustrates the relationship between the NSS and the power spectrum at each frequency bin from 4 to 12 Hz in the medication OFF condition, separately for the left and right STN. In the left STN, a significant cluster between 6 and 10 Hz indicates a significant relationship between increased theta / low-alpha activity and a low neuropsychiatric state. **B** Scatterplot between the NSS and the averaged 6–10 Hz power of the left hemisphere when correcting for the contralateral motor state measured by the hemi-MDS-UPDRS-III score. The values were significantly correlated (Spearman’s rho = −0.77, *p* = 0.002). **C** Shows the relationship between the NSS and the power spectrum at each frequency bin from 4 to 12 Hz in the medication ON condition separately for the left and right STN. No significant relationship was found. NSS neuropsychiatric state score, STN subthalamic nucleus, MDS-UPDRS Movement Disorder Society—Unified Parkinson’s Disease Rating Scale part III.

First, we analyzed the relationship between the NSS and the power at each frequency bin from 4 to 12 Hz separately for the left and the right hemisphere, performing a correlation (Spearman) at each bin in the medication OFF and ON conditions. A significant cluster was found between 6 to 10 Hz (*p* = 0.001) in the medication OFF condition in the left hemisphere, indicating that patients with a low NSS (i.e., predominance of OFF-related neuropsychiatric symptoms/low mood) in the medication OFF state show increased theta/low-alpha activity (Fig. 2A). We further validated this finding using a partial correlation confirming that the relationship between the NSS and the 6–10 Hz spectral power was still significant when controlling for the effect of motor impairment (contralateral hemi-MDS-UPDRS-III) (Spearman’s rho = −0.77, *p* = 0.002) (Fig. 2B). For the right hemisphere, we found a similar trend but no significant effect (Fig. 2A). In the medication ON condition, there was no significant relationship between the NSS and the low-frequency power neither in the left nor right hemisphere (Fig. 2C). In addition, analyses were replicated using the OFF/ON sub-times of the NFS, which showed similar results (Supplementary Fig. 5). Notably, these results were unaffected by clinical parameters such as resting tremor and cognitive performance that can also be represented in the frequency range tested (Supplementary Figs. 6 and 7).

### Neurophysiological correlate of the neuropsychiatric fluctuations

**Fig. 3:**
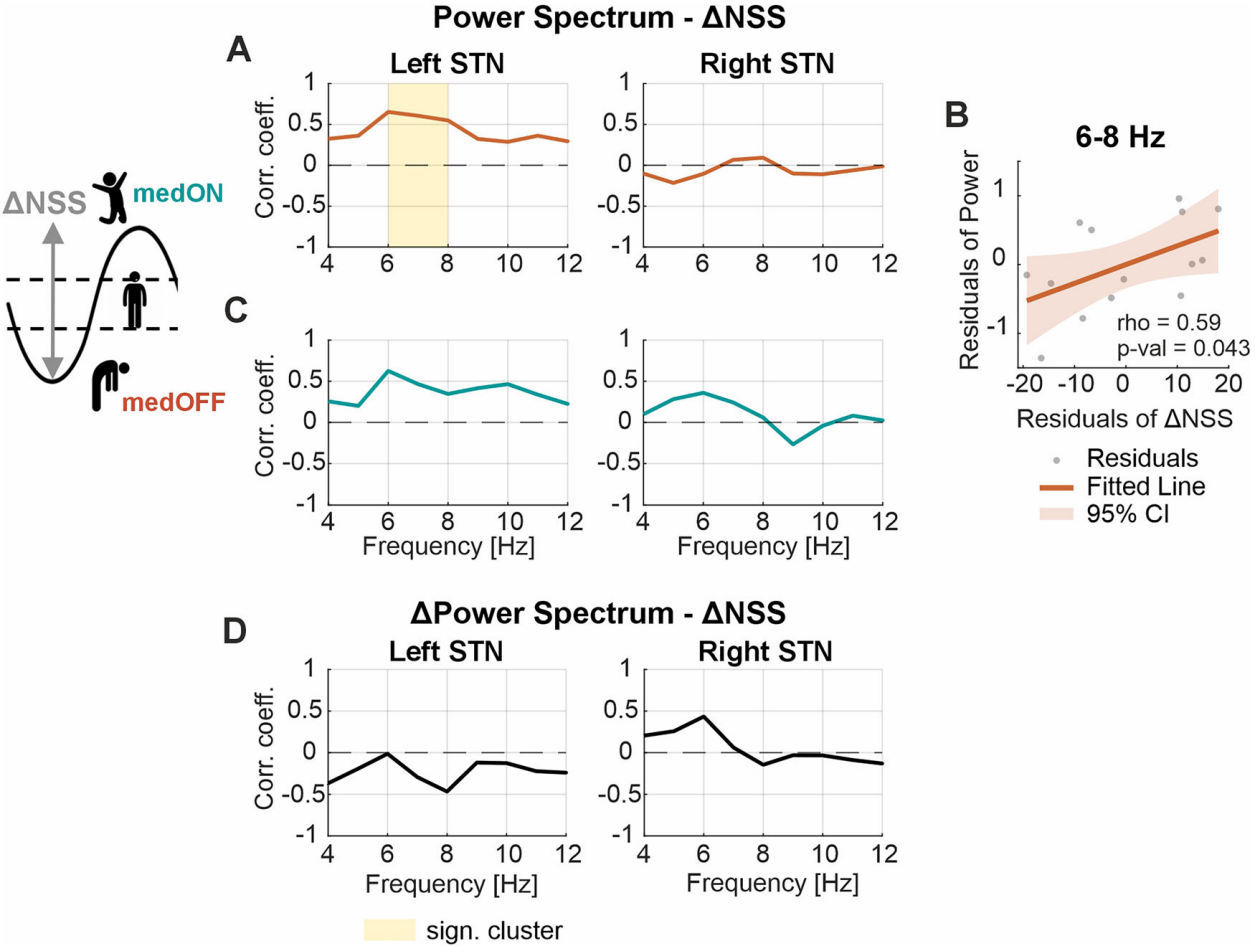
Neurophysiological correlate of neuropsychiatric fluctuations. **A** Illustrates the relationship between the NSS delta (medON–medOFF) and the power spectrum at each frequency bin from 4 to 12 Hz in the medication OFF condition, separately for the left and right STN. In the left STN, a significant cluster between 6 and 8 Hz indicates a significant relationship between increased theta activity and a higher neuropsychiatric state improvement upon medication intake. **B** Scatterplot between the NSS delta and the averaged 6–8 Hz power of the left hemisphere when correcting for the contralateral motor improvement measured by the hemi-MDS-UPDRS-III score, as well as the levodopa dosage. The values were significantly correlated (Spearman’s rho = 0.59, *p* = 0.043). **C** Shows the relationship between the NSS delta and the power spectrum at each frequency bin from 4 to 12 Hz in the medication ON condition separate for the left and right STN. No significant relationship was found. **D** presents the relationship between the NSS delta and the difference in the power spectrum between the medication ON and OFF conditions for each frequency bin from 4 to 12 Hz. No significant relationship was observed. NSS neuropsychiatric state score, STN subthalamic nucleus, MDS-UPDRS Movement Disorder Society—Unified Parkinson’s Disease Rating Scale part III.

Next, we assessed whether ventral low-frequency activity is associated with levodopa-related neuropsychiatric fluctuations. We calculated a fluctuation score based on the NSS delta between the medication OFF and ON conditions and correlated it with the power at each frequency bin from 4 to 12 Hz, separately for the left and the right hemisphere and for the medication OFF and ON conditions. Increased 6 to 8 Hz power in the left hemisphere in the medication OFF condition was significantly positively correlated with the magnitude of neuropsychiatric fluctuations (*p* = 0.036) (Fig. 3A). Correlating the averaged power in the significant frequency range with the fluctuation score when controlling for potential confounds (contra-lateral hemi-MDS-UPDRS-III difference, levodopa dosage) supports the independence of this result of motor impairment and levodopa dosage (Spearman’s rho = 0.59, *p* = 0.043) (Fig. 3B). In the medication ON condition, a similar trend was visible in the left hemisphere, which however did not reach statistical significance (Fig. 3C). No significant relationship was evidenced in the right hemisphere in neither the medication OFF nor ON condition (Fig. 3A, C). Moreover, the change in power upon intake of dopaminergic medication was not significantly correlated with the NSS delta (Fig. 3D). We also assessed the relationship between the spectral data and the chronic neuropsychiatric symptoms (Supplementary Fig. 8), which revealed a slight trend of elevated theta power with a high HADS score for anxiety OFF and ON medication, not reaching statistical significance.

### Translation to chronic stimulation setting

**Fig. 4:**
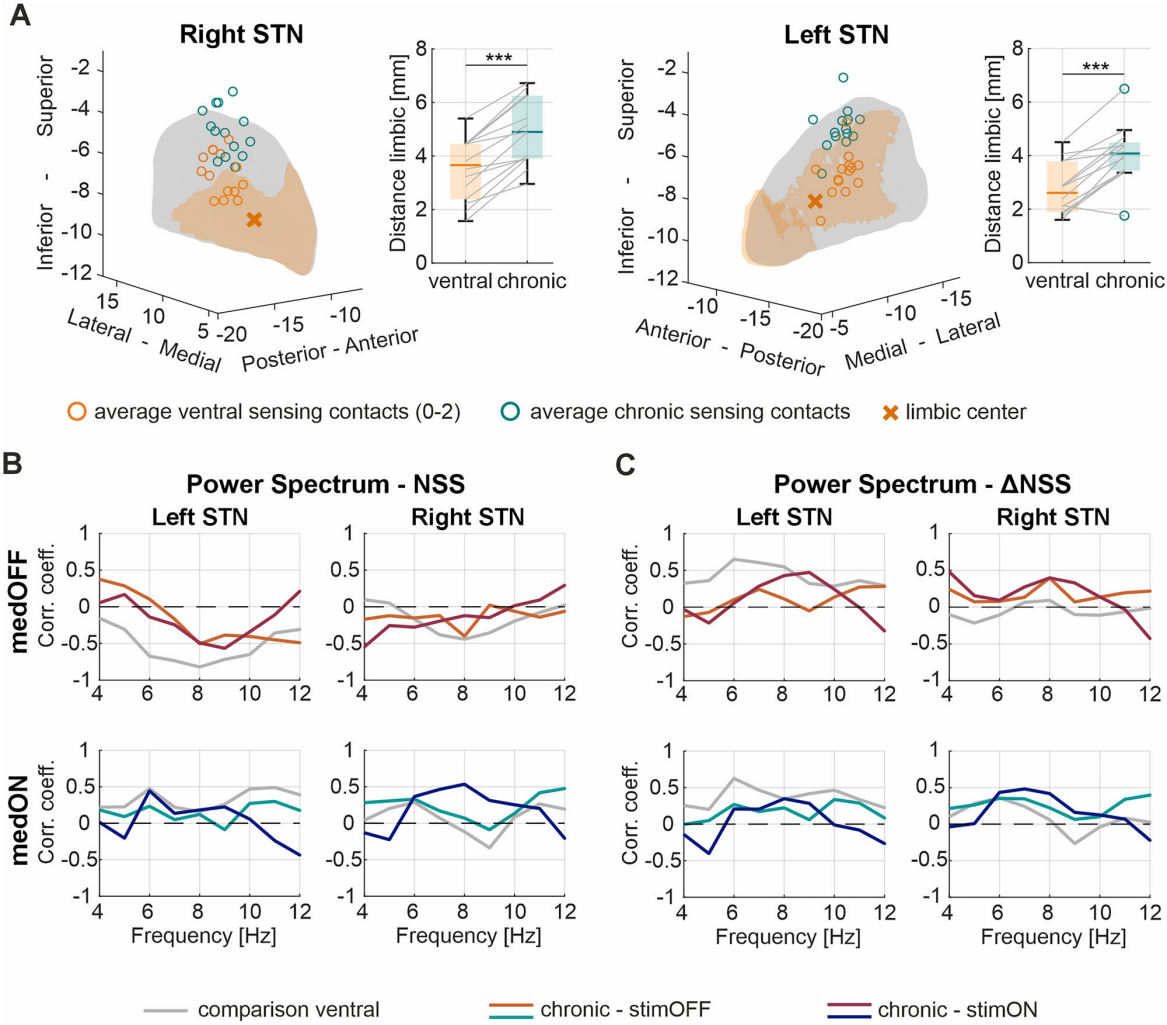
Spectral-anatomical relationship. **A** Shows the average location of the ventral (0–2, orange) and the patient’s individual chronic (0–3 or 1–3, dark green) bipolar sensing configuration of each subject in the STN. The orange shaded area indicates the limbic subregion of the STN and the cross marks its center. The schematic representation of the STN was created using the open-source Lead-DBS toolbox (28). The boxplots represent the Euclidean distance from the average sensing contact location to the limbic center for the ventral (orange) and the patient’s individual chronic configuration (dark green). **B** Illustrates the relationship between the NSS and the power spectrum at each frequency bin from 4 to 12 Hz recorded with the chronic configuration in the left and right STN. Curves in red represent the relationship in the OFF medication and curves in blue in the ON medication condition, while dark red/blue represent the stimulation ON conditions. No significant relationship was found with the chronic setting. The gray curves present the ventral relationship shown in Fig. 2 as comparison. **C** Illustrates the relationship between the NSS delta and the power spectrum at each frequency bin from 4 to 12 Hz recorded with the chronic configuration in the left and right STN. Curves in red represent the relationship in the OFF medication and curves in blue in the ON medication condition. while dark red/blue represent the stimulation ON conditions. No significant relationship was found with the chronic setting. The gray curves present the ventral relationship shown in Fig. 3 as comparison. STN subthalamic nucleus, DBS deep brain stimulation, NSS neuropsychiatric state score.

We tested whether we can capture the above observed spectral-behavioral relationship also when recording with the sensing configuration surrounding the chronically stimulating contacts in both, stimulation OFF and ON conditions. This sensing configuration was in all except one subject further away from the center of the limbic STN compared to the ventral sensing configuration (Fig. 4A and Supplementary Table 1). Comparing the medication effect on the power spectrum between the recordings of the chronic- and the ventral sensing configuration when stimulation is OFF, we did not observe any significant difference (Supplementary Fig. 9). In fact, the trend of a negative correlation between the NSS and the 6–10 Hz power was still present in the left hemisphere using the chronic setting in the OFF medication condition, both with and without stimulation (Fig. 4B). Similarly, the trend of a left-sided positive correlation of the 6–8 Hz activity OFF medication with the neuropsychiatric fluctuation score persisted using the chronic configuration (Fig. 4C). However, they did not reach statistical significance. These results confirm the relevance of the proximity to the limbic STN for capturing neuropsychological informative signals, yet it is encouraging that the trend is preserved when using a more dorsal configuration. Note, for the stimulation ON conditions, the NFS was assessed in both OFF and ON medication, in which all patients improved in the overall neuropsychiatric state upon medication intake (Supplementary Fig. 10A), in parallel to a significant decrease in power between 11–12 Hz in the left STN (*p* = 0.037) (Supplementary Fig. 10B). This power change was not significantly linked with the change in the neuropsychiatric state (Supplementary Fig. 10D). Additionally, we report the effect of stimulation when patients were OFF medication. Stimulation significantly improved the neuropsychiatric state (*n* = 14, *p* < 0.001) (Supplementary Fig. 11A) and led to a significant decrease in the MDS-UPDRS-III (*n* = 14, *p* < 0.001) by 17.6 ± 9.9 points (39 ± 17%) (Table 1 and Supplementary Fig. 2). However, the low-frequency spectrum did not significantly change with stimulation, nor was the stimulation induced spectral modulation (4–12 Hz) related to the NSS delta (Supplementary Fig. 11B, D). Finally, since both beta and gamma activity are typically modulated by both medication and stimulation (Supplementary Fig. 1), we also tested whether these changes were related to the neuropsychiatric metrics, which were not found (Supplementary Fig. 12)^41^.

## Discussion

This work investigates the association between acute neuropsychiatric symptoms and STN oscillations in PD patients chronically implanted with a brain-sense-enabled neurostimulator. We demonstrate that with stimulation turned OFF, elevated theta/low-alpha activity OFF medication is indicative of patients suffering neuropsychiatric OFF-related symptoms and neuropsychiatric fluctuation severity. This spectral-behavioral relationship is most evident for recordings near the limbic STN and is independent of both motor impairment and levodopa dosage. These findings support that sensing-guided treatment strategies in PD may be expanded toward the neuropsychiatric domain.

Neuropsychiatric symptoms and their fluctuations in PD can profoundly affect patients’ quality of life, yet their recognition and treatment remain challenging^1,38,42,43^. This raises the question of whether the management of this symptom domain could benefit from sensing-guided DBS. Ventral STN low-frequency oscillations have emerged as a potential neurophysiological biomarker for neuropsychiatric symptoms^20,22–25,44^. While previous studies primarily associated this spectral feature with chronic symptom traits, our current findings demonstrate that acute hypodopaminergic neuropsychiatric symptoms OFF medication, as well as the magnitude of neuropsychiatric fluctuations following the administration of levodopa, are linked to increased power in the theta/low-alpha range recorded in the OFF medication state. This temporal overlap between neuropsychiatric symptoms and neural oscillations is particularly relevant in the context of sensing-guided DBS, as it may provide immediate input for patient management. Furthermore, the dual association with patients experiencing both pronounced neuropsychiatric OFF symptoms and a stronger psychotropic effect in response to dopaminergic medication is not surprising and may be a consequence of the striatal dopaminergic denervation in mesocorticolimbic pathways, being commonly associated with the intake of higher doses of dopaminergic treatment and thus a higher degree of dopaminergic postsynaptic sensitization of D3–D4 receptors^1,45–50^.

How do the present findings align with previous studies? It is important to interpret them in the context of the clinical neuropsychiatric profiles. Prior studies have linked relatively low ventral alpha power with chronic apathy severity and increased ventral alpha power to trait impulsivity^23,24^. In the current work, we observed increased low-frequency power OFF medication in patients who report more pronounced OFF-related neuropsychiatric symptoms in the medication OFF condition and greater neuropsychiatric fluctuations upon medication intake, which rather matches the clinical profile of trait impulsivity and contrasts with what is typically observed in apathetic patients^51^. In fact, the NSS delta was significantly positively correlated with the QUIP-RS, potentially due to an increased psychotropic effect of medication. This parallel is plausible, as the presence of neuropsychiatric fluctuations was shown to be an independent predictor of impulse control disorders (ICDs) and dopamine dysregulation syndrome, possibly explained by a common mechanism of dopaminergic sensitization^50,52^. The lack of an association of apathy with the change in the acute neuropsychiatric state may be explained by the multidimensionality of apathy and its predominantly chronic temporal dynamics, which differ from the rapid neuropsychiatric fluctuations captured by the NFS^51,53^. In general, how the neuropsychiatric state score correlates with chronic conditions such as ICD or apathy is not yet fully understood and needs further investigation. While the observed spectral-behavioral association was based on the power spectrum in the medication OFF condition, a similar positive trend between the 6–8 Hz power range and the neuropsychiatric fluctuation score was also evidenced ON medication. This finding echoes a previous report of increased STN theta activity in PD patients with ICDs ON medication^22^. Thus, elevated theta/alpha activity may serve as a potential biomarker reflecting both acute hypo-dopaminergic symptoms and the degree of neuropsychiatric fluctuations, possibly further extending along a continuum toward ICDs^45^. The lack of a relationship between the STN low-frequency activity and the neuropsychiatric state in the ON medication condition is of interest and could be due to the design of the NFS, as it was shown to be more sensitive to OFF-related symptoms^37^. Alternatively, the relationship is indeed attenuated in the dopamine replaced state, potentially due to the modulatory effect of medication on theta activity, which is not observed with stimulation alone^30–33^.

A recurring question is whether brain activity patterns involved in neuropsychiatric symptom encoding exhibit lateralization. In the present study, we observed a stronger clinical–spectral relationship in signals from the left STN. However, whether this reflects a true intrinsic functional lateralization remains unclear, as our cohort also showed subtle anatomical differences with left-sided electrodes positioned slightly more ventrally, closer to the limbic STN. Thus, consistent with our overarching hypothesis of spectrally profiling limbic activity, the observed lateralization may be attributable to anatomical factors rather than functional asymmetry. Additionally, the existing literature on neuropsychiatric lateralization in the STN remains inconclusive, making it difficult to contextualize our findings within a broader framework^19,22–24,26–29^. Studies linking STN activity with chronic neuropsychiatric symptoms have not reported any lateralization of biomarkers^22–24,29^. In contrast, immediate neurophysiological responses to emotional stimuli appear stronger in the right STN^26–28^, whereas stimulation of the left STN has been associated to improvements in mood and anxiety^54–56^. Hence, dedicated study designs with unilateral stimulation at different frequencies and locations, together with the spectral profile, could help to systematically approach this topic.

Having demonstrated that routinely implanted DBS leads can capture neurophysiological signals associated with the limbic subregion of the STN, it is important to consider how this insight can be translated into clinical practice. Sensing-guided DBS strategies span from assisted programming to real-time adaptive stimulation. Our findings support the potential utility of incorporating low-frequency activity into the DBS programming workflow, possibly informing decisions about whether to avoid or deliberately target limbic areas depending on individual clinical goals^57^. While existing evidence suggests a contingency of electrode placement and frequency, still little is known about a systematic clinical effect and stimulation parameters required for purposefully modulating limbic circuits^54–56,58–60^. Moreover, since dopaminergic medication exposure and receptor sensitization are key factors influencing chronic neuropsychiatric conditions, we see further clinical potential in using chronic sensing of limbic activity to track longitudinal changes in the neuropsychiatric–spectral relationship. This could help clinicians identify patients at risk of developing impulse control and related disorders, including dopamine dysregulation syndrome or apathy, allowing timely therapeutic adjustments by rebalancing medication and stimulation to prevent the emergence of these disabling complications over time. Ultimately, the goal is to develop integrative, multi-modal adaptive DBS systems that can simultaneously account for multiple symptom domains, each with distinct temporal dynamics and spatial representation within the DBS target^61,62^. While adaptive DBS approaches for the motor domain have already been conceptualized, experimentally validated, and, in some cases, translated into clinical practice, robust evidence supporting similar strategies for the neuropsychiatric domain is still lacking. We consider the present study an important step toward this vision, as it links acute neuropsychiatric symptoms to STN neural activity. However, in contrast to neurophysiological motor biomarkers such as beta activity, which scales with motor states in both the OFF and ON medication conditions, theta activity in our cohort only scales with the neuropsychiatric state in the OFF-medication state. This limitation constrains to fully capture neuropsychiatric fluctuation at this stage. Future work should therefore aim to characterize the precise temporal dynamics of this biomarker in daily life as well as over longer time scales and show how repeated repetitive medication intakes and neuromodulation influences the neuropsychiatric and neurophysiologic profile^41,63,64^.

The explorative and pilot nature of this study, together with the small sample size, may limit the robustness and generalizability of these findings. Also, since the data were derived as part of the clinical routine pipelines, the medication and stimulation conditions were not randomized. Possible overlapping effect of medication and stimulation cannot be excluded, but was minimized by following a well-established levodopa challenge protocol, which resulted in expected clinical states. Despite this, the present result for the acute neuropsychiatric state can be rationally linked to previous observations on chronic biomarkers of levodopa-responsive symptoms along the apathy-impulsivity spectrum. In real-life conditions, however, with regular medication intake, long-lasting and overlapping pharmacological effects would occur, which may influence the presently shown clinical–spectral relationship. Moreover, DBS leads were anatomically implanted to primarily treat motor symptoms, thus, the typical more ventral limbic activity was rather recorded from a distance and stimulation effects were only investigated for the patient's individual chronic setting. Therefore, we assume that the relationship between brain activity and neuropsychiatric symptoms could have been underestimated in this work.

These findings advance our understanding of the relationship between STN activity and the acute neuropsychiatric symptom state in PD. Future research should investigate how low-frequency activity, as well as other frequency bands, are modulated by different stimulation paradigms and how their dynamics evolve across medication cycles and over longer time periods in parallel with changes in the neuropsychiatric profile. Overall, our work encourages expanding sensing-guided DBS strategies to incorporate multiple symptom domains, moving beyond motor control toward a more holistic approach to patient care.

## Methods

### Patients

In this study, we screened PD patients with bilateral STN DBS implanted at the University Hospital in Bern with a Percept^TM^ PC (Medtronic, MN, USA), who underwent a clinical and neurophysiological assessment in the 4 possible combinations of dopaminergic medication and stimulation approximately one year after DBS surgery as part of the clinical routine. Patients were excluded from the study if they had a Montreal Cognitive Assessment (MoCA) score below 20 points to ensure reliable responses in the self-administered questionnaires. From 03/2023 to 07/2025, we identified 16 patients, of whom two patients were excluded: One subject had a MoCA score of 17/30 points, and a second subject did not respond to the administered levodopa dosage, showing a worsening of motor symptoms, as well as frequent dozing off during the LFP recordings. The final cohort included a total of 14 patients (2 females), all receiving DBS primarily for motor fluctuations. Among those thirteen classified as akinetic-rigid subtype and one as mixed subtype PD, with an average age of 62 years (range 39–74), and an average disease duration of 12 years (range 6–25) at the time of the assessment, which took place on average 12 months (range 11–16) after DBS surgery (Table 1). All subjects have signed the general consent for further usage of their clinical data for scientific purposes. The study involving human participants was approved by the local ethics committee of Bern (2017-00551) and adhered to the ethical standards of the Declaration of Helsinki.

### DBS surgery

All subjects underwent DBS surgery under general anesthesia, with the procedure recently detailed^65^. Briefly, the DBS target was identified on the T2-sequence of the preoperative 3-T magnetic resonance imaging (MRI), co-registered with preoperative stereotactic computed tomography (CT) scans acquired using the Leksell G frame. Image fusion and trajectory planning were performed with Brainlab Elements software (Brainlab AG, Munich, Germany). Intraoperative targeting was optimized using microelectrode recordings and selective intraoperative test stimulation to determine thresholds for capsular side-effects.

### Clinical assessment

An acute levodopa challenge was performed after withdrawal of dopamine agonist for more than 48 h and of levodopa formulations for more than 12 h, in order to minimize lasting medication effects. Since data were derived from clinical routine, the assessment followed a standardized approach and always started in the morning with the following order of conditions: ON stimulation/OFF medication, OFF stimulation/OFF medication, OFF stimulation/ON medication, and ON stimulation/ON medication. The target levodopa dose for the assessment corresponded to 1.5 times the patients’ usual morning dose, with adjustments depending on patients’ clinical state, administered as a liquid formulation of levodopa plus benserazide. The medication ON condition started when the patient and examiner agreed that a stable medication ON state was reached, which was on average 50 min (ranging from 35 to 80 min) after medication intake. After stimulation was switched OFF and ON, the motor assessment started after around 15 min, the neuropsychiatric assessment after a minimum of 30 min, followed by the LFP recording. First, the motor state was quantified using the Movement Disorder Society—Unified Parkinson’s Disease Rating Scale part III (MDS-UPDRS-III), followed by the neuropsychiatric state evaluation using the self-assessed NFS. The NFS has been validated in French, used in multiple studies, with a larger multi-centric validation including multiple languages ongoing^35–39^ (NCT04366804). The NFS provides two sub-scores, ten ON-items reflecting typical ON symptoms and ten OFF-items reflecting typical OFF symptoms in PD. Patients rated each item on a scale from 0 (does not describe how I feel right now) to 3 (describes a lot of how I feel right now), resulting in a maximal total score of 30 points each^35^. The single items can be found in the Supplementary Table 2. The sub-scores can be combined to a composite score, the Neuropsychiatric State Score (NSS)^38^:

1 $$NSS=Score\,ON\,items+(30-Score\,OFF\,items)$$

The NSS ranges from 0 to 60, with 0 corresponding to a predominance of OFF-related neuropsychiatric symptoms (low mood) and 60 to a predominance of ON-related neuropsychiatric symptoms (elevated mood).

To quantify the neuropsychiatric fluctuations between treatment conditions, we calculated the difference between the NSS in the ON and OFF medication state:

2 $$\Delta NSS=NS{S}_{ON}-NS{S}_{OFF}$$

In a separate appointment with a neuropsychologist, the patient’s chronic neuropsychiatric condition was assessed using the MoCA, the QUIP-RS, the HADS, and the Starkstein Apathy Scale. The scores of the clinical assessments can be found in the Supplementary Tables 3 and 4.

### Neurophysiological recordings

In each condition, bilateral STN-LFPs were recorded wirelessly with a sampling rate of 250 Hz and the patient at rest, seated comfortably and eyes open for 2 minutes. In all conditions, the “Brain Sense Streaming” mode was used, with the sensing contacts surrounding the contacts used for chronic stimulation. If the chronic stimulation setting was not compatible with sensing, the setting was adjusted (in 2 out of 14 patients; see Table 1). In the stimulation OFF conditions, LFPs were additionally recorded in the “Brain Sense Survey Indefinite Streaming” mode allowing to record simultaneously with multiple bipolar sensing configurations: 0–3, 1–3, and 0–2 (Fig. 1A). Note, all recording settings were used as they are commercially enabled in the Medtronic Percept PC/RC. Current technical limitations of in-built systems were considered in the study design and signal processing^66–68^.

### Signal processing

All data were analyzed offline using MATLAB (version R2023b; The MathWorks, Inc., Natick, Massachusetts). Signals were band-pass filtered from 0.5 to 105 Hz using a fourth-order Butterworth filter. Each filtered signal was inspected for and if necessary cleared from heart rate artefacts using the *perceive_ecg* function (Perceive toolbox)^66^. The signals were decomposed into frequency components of 1 Hz resolution using the Morlet Wavelet transform (*ft_specest_wavelet*, width = 10, gwidth = 5; Fieldtrip, Donders Institute for Brain, Cognition and Behaviour, 2010). The power spectral density of each signal was normalized within the respective conditions by subtracting the mean power across the reference frequency range (4–45 Hz and 55–90 Hz) and dividing by the corresponding SD. For the analyses comparing the power spectra between two treatment conditions, the normalization procedure was applied on the concatenated power spectra of the respective conditions. All normalized power values within the frequency range of interest described below can be found in the Supplementary Tables 5 and 6.

### Localization of DBS contacts

DBS contacts were localized using the Lead-DBS MATLAB toolbox (version 2.6)^69^. Preoperative MRI and postoperative CT scans were co-registered, corrected for brain shift and normalized to the Montreal Neurological Institute (MNI) space (MNI152 NLIN 2009b). The electrode trajectory and position were reconstructed semi-automatically and corrected if necessary^70^. The source of the bipolar signal was approximated by averaging the sensing contacts’ location and further visualized and related to the DISTAL atlas^21^.

### Spectral-behavioral analysis

All statistical analyses were performed using MATLAB. Results are presented as mean ± SD, unless otherwise stated. The clinical scores between two conditions, as well as the recording positions between the left and right hemispheres and between ventral and chronic recording settings were compared using a Wilcoxon signed-rank test. For all spectral-behavioral analysis, we considered the low-frequency range from 4 to 12 Hz with a bin size of 1 Hz for the left and right STN separately^22–28,44^. We primarily considered the ventral broad bipolar sensing configuration (0–2) due to its proximity to the limbic STN and potential compatibility as adaptive DBS contact-configuration set-up. However, we also repeated the analyses for the sensing configuration surrounding the chronically stimulating contacts. To assess the power spectral density difference in the low-frequency range (4–12 Hz) between two treatment conditions, as well as between the left and right hemispheres within each condition, a cluster-based permutation was performed. For each frequency bin, we computed the power difference. A permutation distribution was generated by randomly permuting the sign of the power difference over the frequency bins of interest ranging from 4 to 12 Hz (1 Hz resolution) for a subset of subjects (*n* = 14) 5000 times. For each frequency bin, the z-statistic (of the actual mean power difference) was computed based on the distribution of the 5000 permutations. Using an alpha of 0.05 as cluster-building threshold, the resulting cluster, consisting of all frequency points that exceeded the initial threshold, were compared against the probability of clusters occurring by chance within the permutation distribution. Only clusters in the observed data that were larger than 95% of the distribution of cluster obtained in the permutation analysis were considered significant^71^. With this approach, we control for the family-wise error rate by assessing the significance of clusters as a whole rather than at individual points. The relationship between the NSS and NSS delta and the power at each frequency bin in the range of interest was analyzed across 14 subjects using Spearman correlation separately for the left and right hemisphere. The correlation coefficients over the defined frequency range were tested for significance using the above described cluster-based permutation procedure. Here, a permutation distribution was derived by shuffling the order of the scores 5000 times and calculating the correlation with the power values for each permutation. At the frequency bins within a significant cluster, the power values were averaged and a partial correlation (Spearman) was performed with the NSS/NSS delta to control for the respective contralateral hemi-MDS-UPDRS-III score. For the ON-medication state statistics, the levodopa dosage administered was additionally controlled for to rule out a potential confounding effect.

## Supplementary information

Supplementary Information

## Data Availability

All data used to derive the main results are provided in the supplementary material. Neurophysiological raw data and imaging data are subject to data sharing agreements and are only available upon reasonable request from the corresponding author, gerd.tinkhauser@insel.ch.

## Code availability

The underlying code for this study is not publicly available but may be made available to qualified researchers on reasonable request from the corresponding author, gerd.tinkhauser@insel.ch.
