# Supplementary material for: Low-frequency activity in the subthalamic nucleus informs about the acute neuropsychiatric state in Parkinson’s disease
Source: NPJ Parkinsons Dis. 2026 Jan 15;12:21. doi: 10.1038/s41531-025-01233-3 (PMC12820131; doi:10.1038/s41531-025-01233-3)
Supplement: Supplementary file 1 — Supplementary Information [file 41531_2025_1233_MOESM1_ESM.pdf]

Low Frequency Activity in the Subthalamic Nucleus informs about the Acute Neuropsychiatric State in Parkinson's disease

Supplementary material

Results

	Left				Right			
**Subject**	**Configuration**	**X**	**Y**	**Z**	**Configuration**	**X**	**Y**	**Z**
1	chronic (1-3)	-14.61	-10.91	-3.26	chronic (1-3)	11.67	-15.14	-3.66
	ventral (0-2)	-13.99	-11.88	-5.19	ventral (0-2)	10.94	-15.67	-5.78
2	chronic (1-3)	-12.69	-14.52	-4.57	chronic (1-3)	11.01	-13.65	-5.97
	ventral (0-2)	-11.95	-15.17	-6.57	ventral (0-2)	10.20	-14.46	-7.90
3	chronic (1-3)	-12.64	-12.17	-5.47	chronic (1-3)	13.34	-12.67	-5.13
	ventral (0-2)	-11.97	-13.20	-7.49	ventral (0-2)	12.63	-13.75	-7.11
4	chronic (1-3)	-13.26	-10.76	-6.32	chronic (1-3)	11.86	-10.77	-5.61
	ventral (0-2)	-12.68	-11.89	-8.35	ventral (0-2)	11.15	-11.99	-7.49
5	chronic (1-3)	-12.21	-14.38	-5.32	chronic (1-3)	12.83	-13.46	-4.75
	ventral (0-2)	-11.45	-15.65	-7.29	ventral (0-2)	12.26	-14.75	-6.74
6	chronic (1-3)	-12.88	-13.15	-4.36	chronic (1-3)	13.68	-12.11	-3.84
	ventral (0-2)	-12.20	-14.00	-6.76	ventral (0-2)	12.88	-13.25	-5.92
7	chronic (1-3)	-12.72	-12.17	-5.65	chronic (1-3)	12.40	-11.01	-6.37
	ventral (0-2)	-11.77	-13.45	-7.51	ventral (0-2)	11.44	-12.22	-8.29
8	chronic (1-3)	-12.93	-12.95	-5.86	chronic (0-3)	11.16	-12.69	-6.57
	ventral (0-2)	-12.09	-13.79	-8.02	ventral (0-2)	10.69	-13.11	-7.66
9	chronic (1-3)	-10.68	-13.00	-7.05	chronic (1-3)	12.72	-11.51	-5.90
	ventral (0-2)	-9.82	-13.78	-9.09	ventral (0-2)	12.01	-12.63	-7.88
10	chronic (1-3)	-12.75	-13.13	-5.20	chronic (1-3)	11.76	-13.74	-6.30
	ventral (0-2)	-12.00	-14.22	-6.95	ventral (0-2)	10.87	-14.77	-8.00
11	chronic (1-3)	-13.63	-11.83	-5.08	chronic (1-3)	12.22	-11.06	-4.65
	ventral (0-2)	-12.85	-12.64	-7.18	ventral (0-2)	11.67	-12.14	-6.70
12	chronic (0-3)	-11.37	-14.69	-5.03	chronic (0-3)	11.10	-13.63	-4.80
	ventral (0-2)	-10.96	-15.27	-6.00	ventral (0-2)	10.76	-14.30	-5.72
13	chronic (1-3)	-12.14	-10.62	-4.96	chronic (1-3)	13.45	-11.03	-3.37
	ventral (0-2)	-11.57	-11.50	-7.15	ventral (0-2)	12.85	-11.88	-5.55
14	chronic (1-3)	-13.73	-12.28	-4.98	chronic (1-3)	13.29	-12.62	-3.74
	ventral (0-2)	-13.03	-13.37	-7.06	ventral (0-2)	12.49	-13.53	-5.86

Supplementary table 1. **MNI coordinates of bipolar signal source.** The coordinates (x, y, z) are calculated by averaging the sensing contacts' location part of either the ventral (0-2) or patient individual chronic sensing configuration.

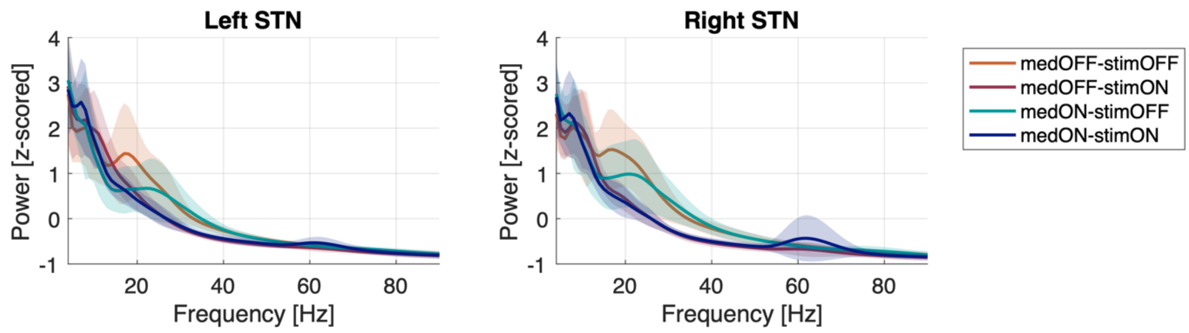

Supplementary figure 1. **Medication and Stimulation effect on power spectrum.** Shows the power spectrum from 4-90 Hz averaged across all subjects ±SD in the medication and stimulation OFF and ON conditions of the left and right STN using the patient individual chronic configuration. STN, subthalamic nucleus; SD, standard deviation.

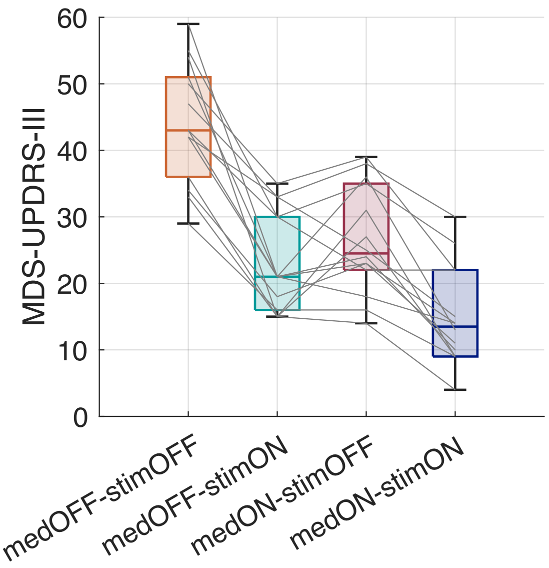

Supplementary figure 2. **MDS-UPDRS III scores.** Total score of the MDS-UPDRS-III in the medication / stimulation OFF/ON conditions. UPDRS, Movement Disorders Society - Unified Parkinson’s Disease Rating Scale part 3.

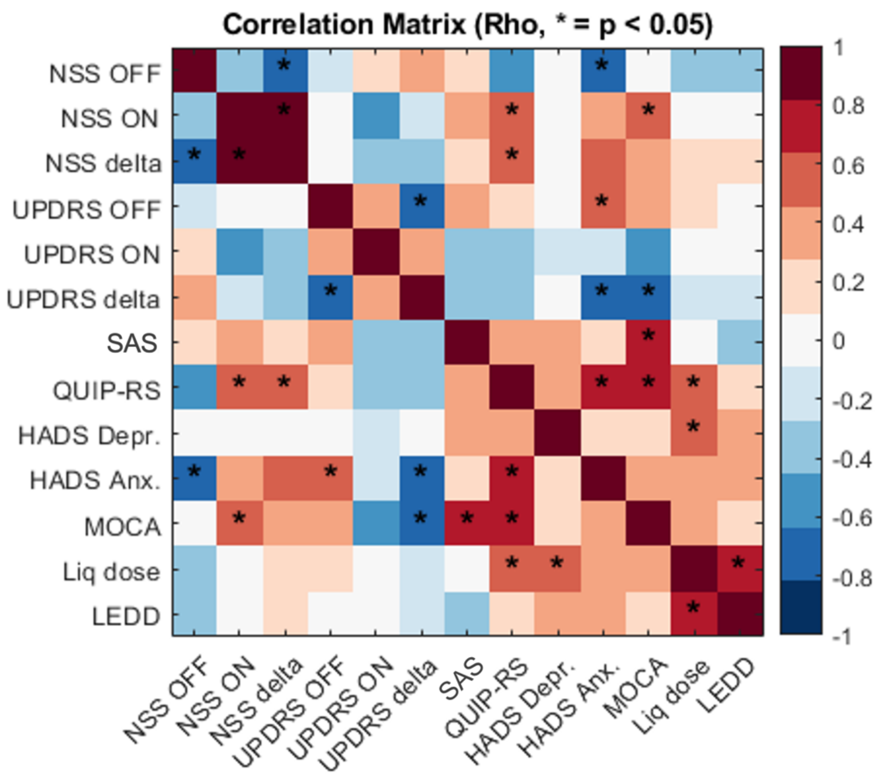

Supplementary figure 3. **Correlation matrix of clinical scores**. The colors of the correlation matrix index the correlation coefficient when correlating (Spearman) two scores with each other across the cohort. OFF and ON correspond to the medication condition, while stimulation was OFF. A star marks significant (p-value<0.05) correlations. NSS, neuropsychiatric state score; UPDRS, Movement Disorders Society - Unified Parkinson’s Disease Rating Scale part 3; QUIP-RS, Questionnaire for Impulsive-Compulsive Disorders in Parkinson’s Disease – Rating Scale; HADS, Hospital Anxiety and Depression Scale; Depr, Depression; Anx, Anxiety; SAS, Starkstein Apathy Scale; MOCA, Montreal Cognitive Assessment; LEDD, levodopa equivalent daily dose.

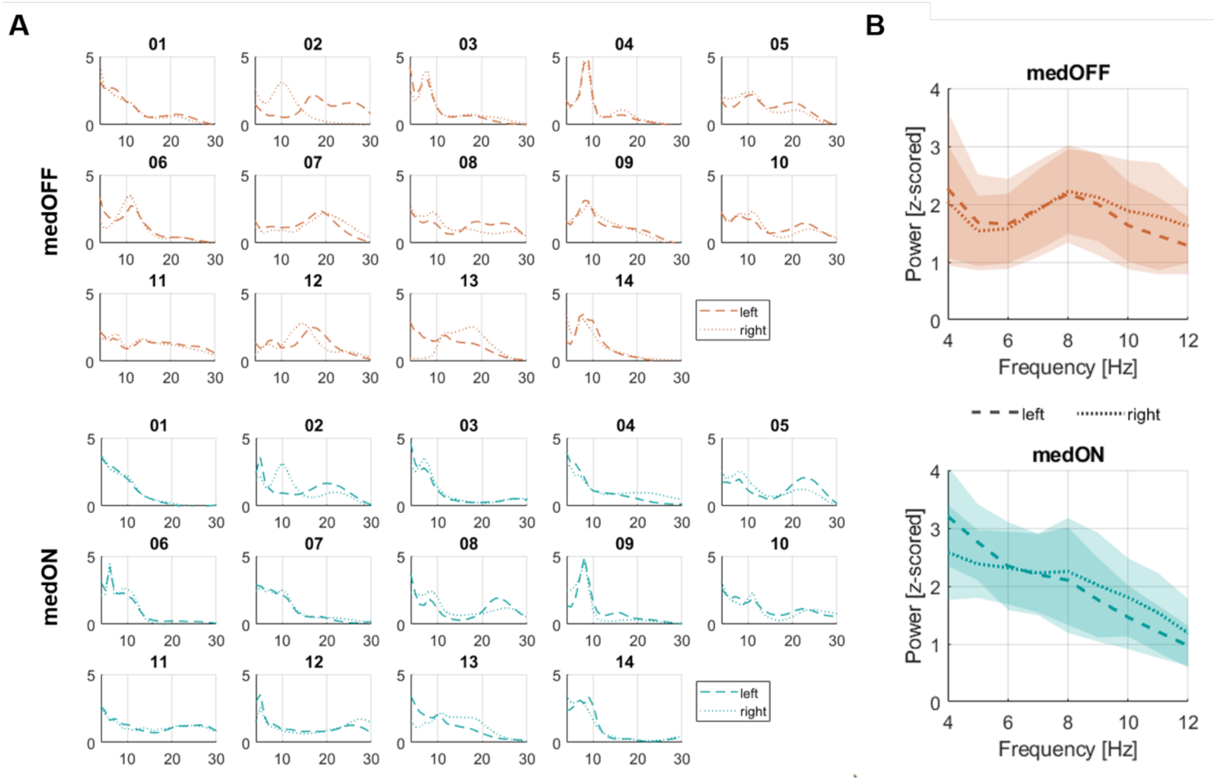

Supplementary figure 4. **Left vs. right power spectrum. A.** Illustration of the power spectrum of each single subject measured with the ventral configuration (0-2) in the left and right STN OFF (red) and ON (blue) medication. **B.** Shows the average power spectrum across the cohort ±SD in the left and right STN in the OFF (red) and ON (blue) medication condition. No significant difference was seen between the left and right STN neither in the OFF nor ON medication condition. STN, subthalamic nucleus.

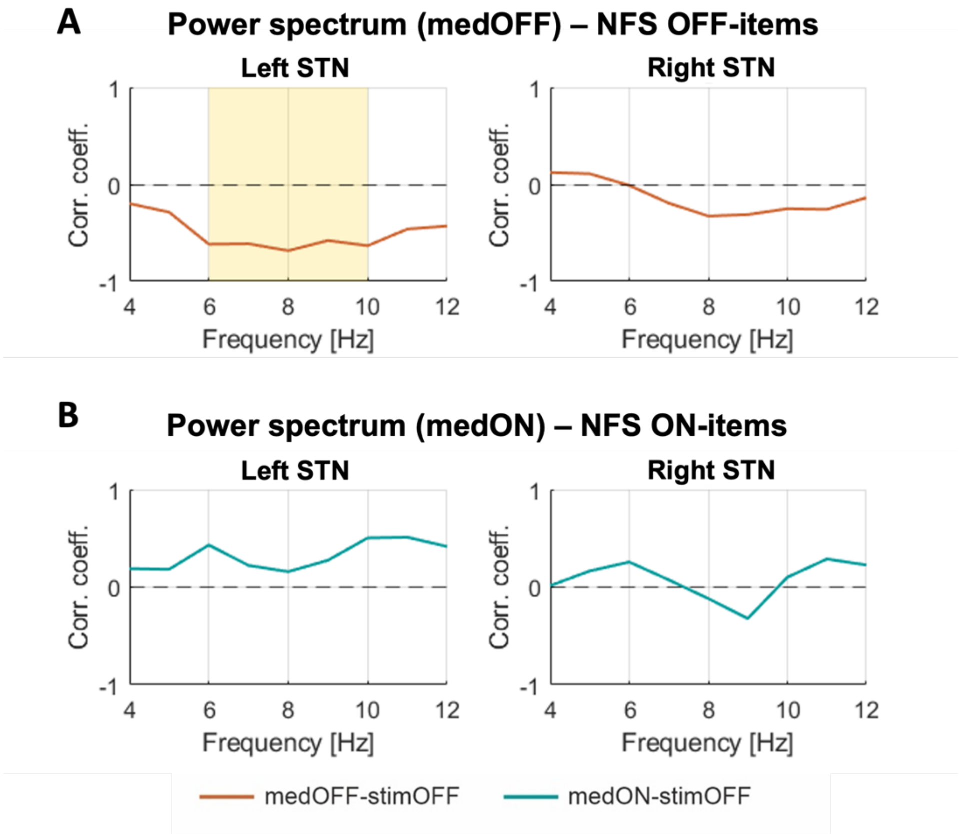

Supplementary figure 5. **Neurophysiological correlate of the NFS sub-item. A.** Illustrates the relationship between the NFS OFF-items and the power spectrum at each frequency bin from 4-12 Hz in the medication OFF condition, separately for the left and right STN. In the left STN, a significant cluster between 6 and 10 Hz indicates a significant relationship between increased theta / low-alpha activity and a low neuropsychiatric state. **B.** Shows the relationship between the NFS ON-item and the power spectrum at each frequency bin from 4-12 Hz in the medication ON condition separately for the left and right STN. No significant relationship was found. NSS, neuropsychiatric state score; STN, subthalamic nucleus.

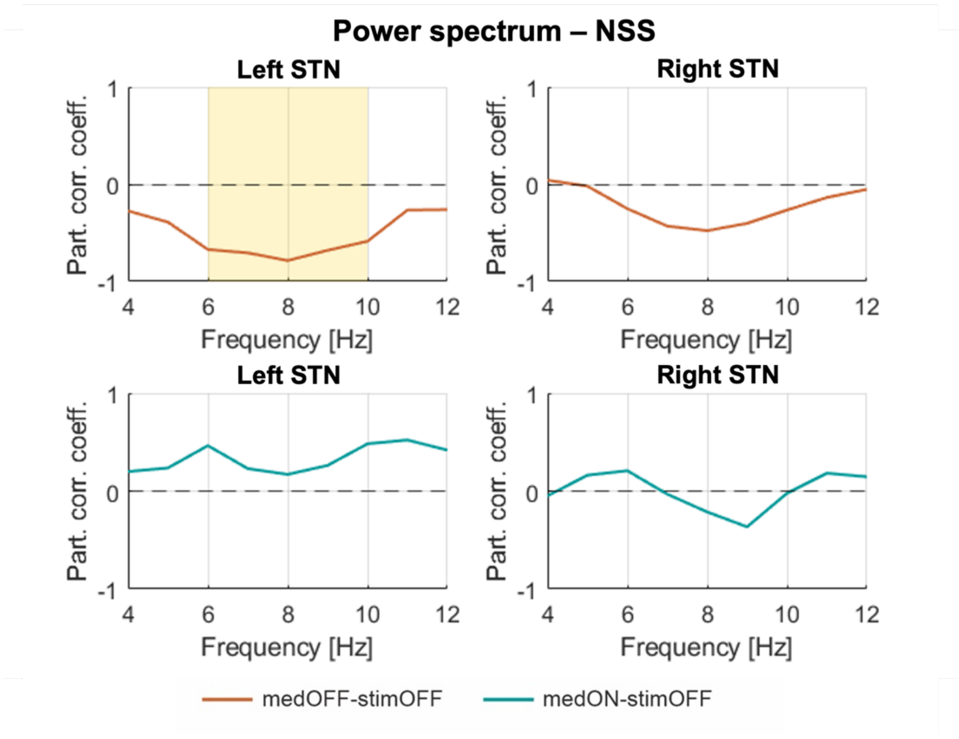

Supplementary figure 6. **Neurophysiological correlate of the acute neuropsychiatric state when correcting for resting tremor.** Illustrates the partial correlation between the NSS and the power spectrum at each frequency bin from 4-12 Hz in the medication OFF (red) and ON (blue) condition, separately for the left and right STN when correcting for the hemi-lateral resting tremor based on the MDS-UPDRS-III score. In the left STN OFF medication, a significant cluster between 6 and 10 Hz indicates a significant relationship between increased theta / low-alpha activity and a low neuropsychiatric state. ON medication, no significant relationship was found. NSS, neuropsychiatric state score; STN, subthalamic nucleus; MDS-UPDRS: Movement Disorder Society - Unified Parkinson's Disease Rating Scale part III.

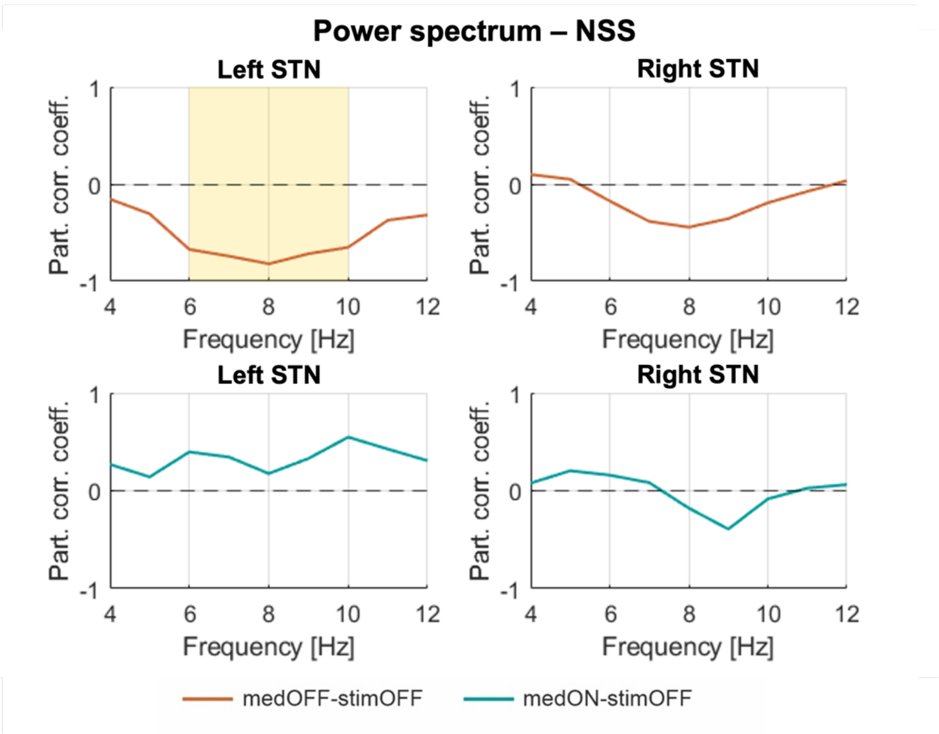

Supplementary figure 7. **Neurophysiological correlate of the acute neuropsychiatric state when correcting for cognition.** Illustrates the partial correlation between the NSS and the power spectrum at each frequency bin from 4-12 Hz in the medication OFF (red) and ON (blue) condition, separately for the left and right STN when correcting for the cognition based on the MoCA score. In the left STN OFF medication, a significant cluster between 6 and 10 Hz indicates a significant relationship between increased theta / low-alpha activity and a low neuropsychiatric state. ON medication, no significant relationship was found. NSS, neuropsychiatric state score; STN, subthalamic nucleus; MOCA, Montreal Cognitive Assessment.

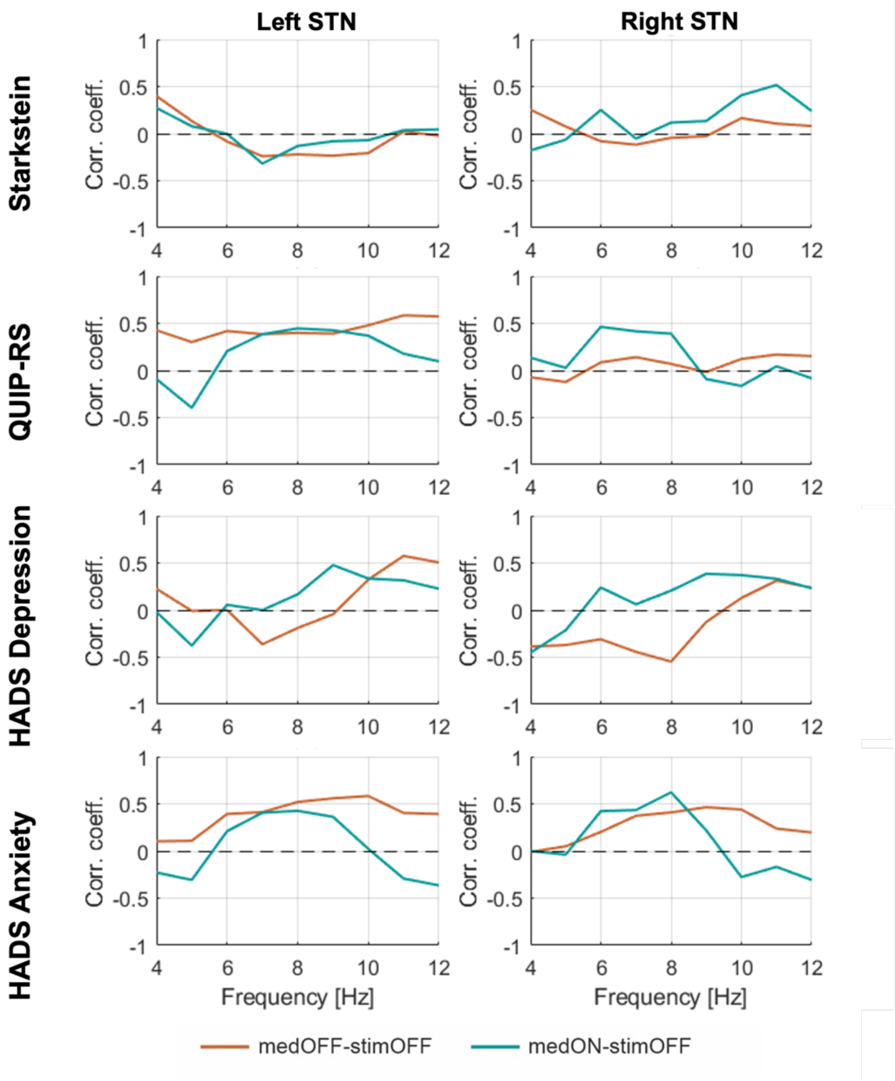

Supplementary figure 8. **Neurophysiological correlate of chronic neuropsychiatric symptoms**. Illustrates the relationship between the scores of four neuropsychiatric scales and the power spectrum at each frequency bin from 4-12 Hz in the medication OFF (red) and ON (blue) condition, separately for the left and right STN. No significant relationship was found. QUIP-RS, Questionnaire for Impulsive-Compulsive Disorders in Parkinson's Disease – Rating Scale; HADS, Hospital Anxiety and Depression Scale.

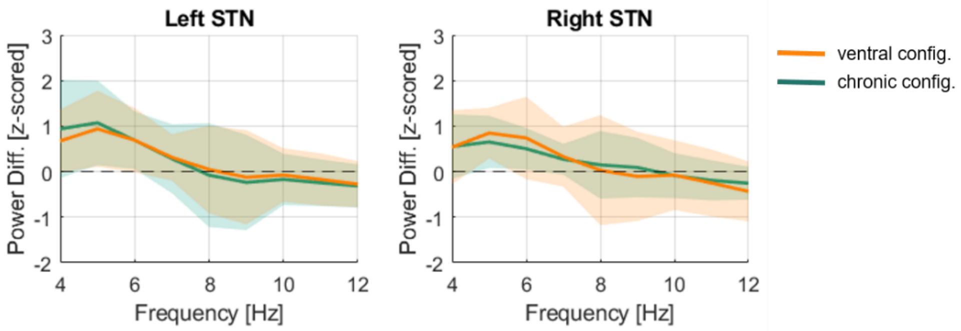

Supplementary figure 9. **Medication effect on power spectrum of the ventral vs. chronic configuration.** Illustrates the average difference in spectral power from 4-12 Hz between the medication ON and OFF condition (ON-OFF) across all subjects ±SD when recorded with the ventral (orange) versus the patient individual chronic configuration (dark green) in the STN. No significant difference was observed. STN, subthalamic nucleus. config, sensing configuration.

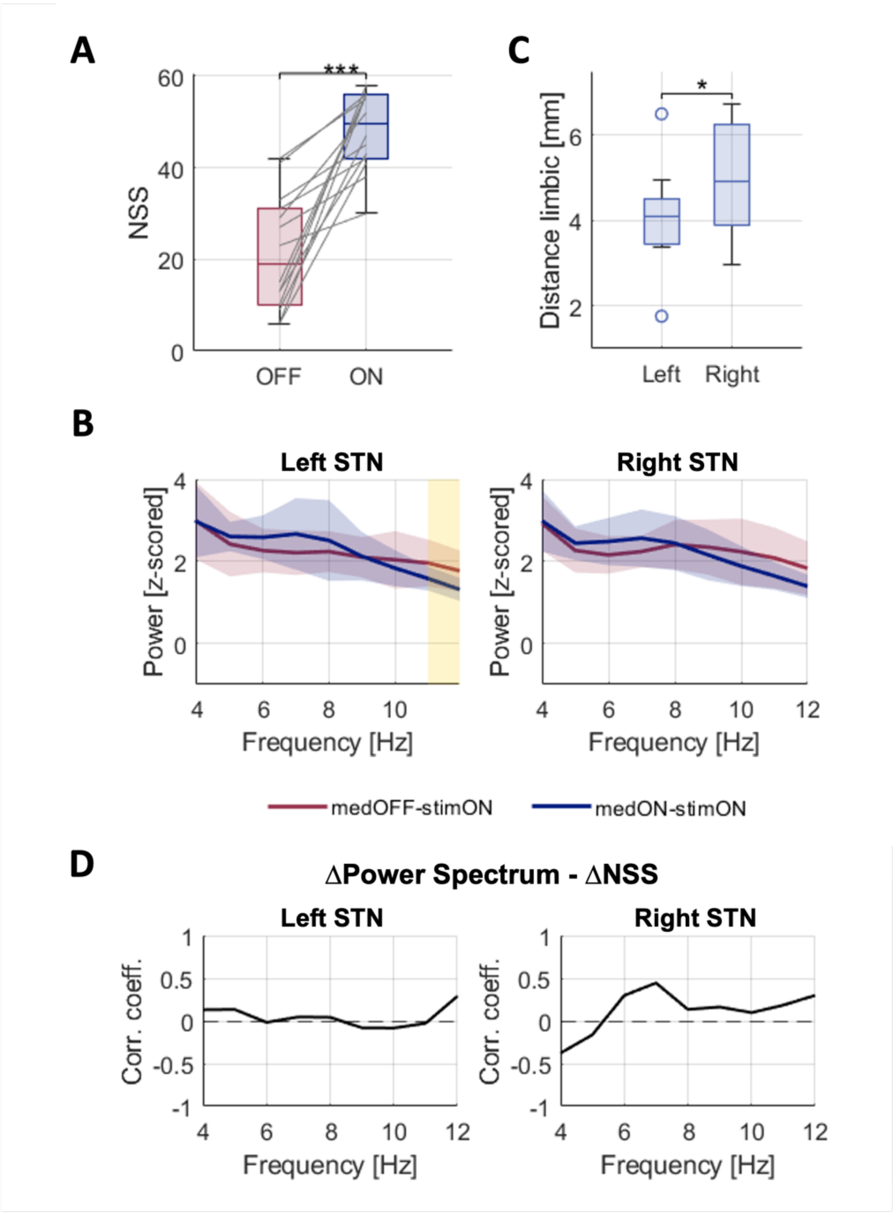

Supplementary figure 10. **Medication effect on neuropsychiatric state and STN neurophysiology ON stimulation. A.** The boxplot presents the distribution of the NSS of all subjects in the medication OFF(red) and ON (blue) conditions (ON stimulation). **B.** Shows the power spectrum from 4-12 Hz averaged across all subjects ±SD in the medication OFF and ON condition when stimulation is ON. The shaded area (yellow) indicates a significant difference in power. **C.** Illustrates the Euclidean distance from the average sensing contact location using the chronic configuration to the centroid of the limbic STN in the left and right hemisphere. **D.** Presents the relationship between the NSS delta and the difference in the power spectrum between the medication ON and OFF condition while stimulation was ON for each frequency bin from 4-12 Hz. No significant relationship was observed. STN, subthalamic nucleus; LFP, local field potentials; DBS, deep brain stimulation; NSS, neuropsychiatric state score; SD, standard deviation.

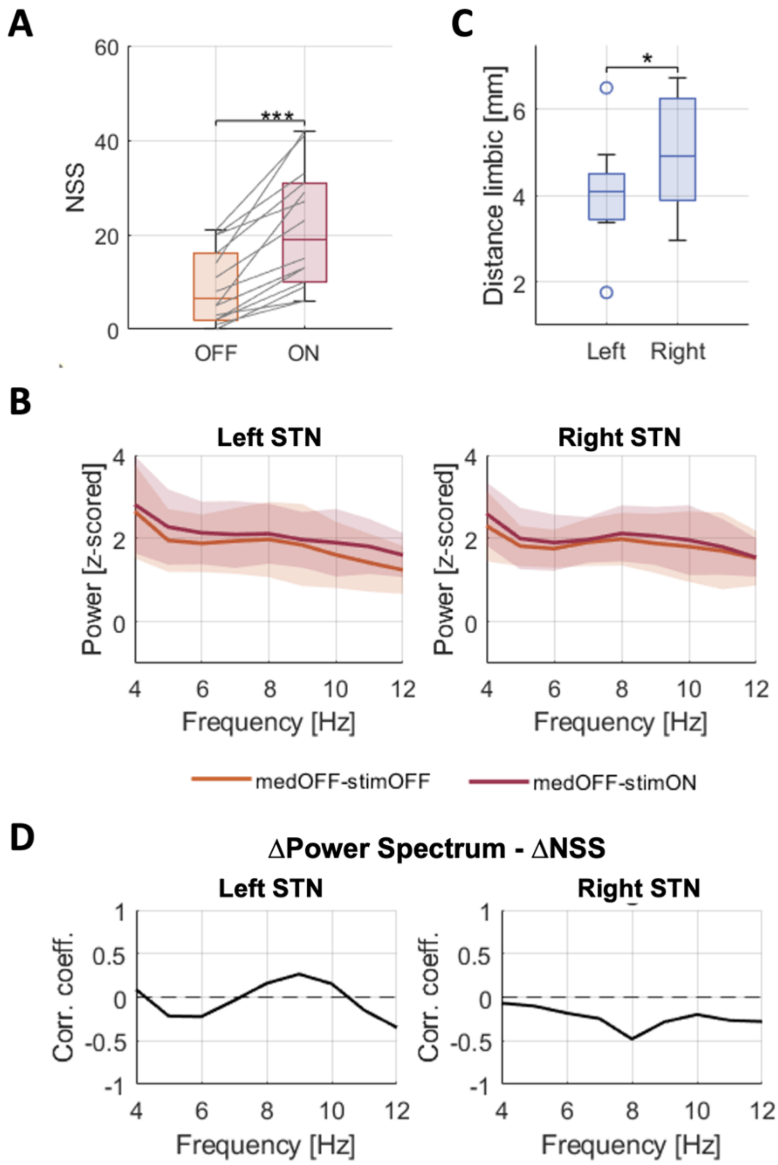

Supplementary figure 11. **Stimulation effect on neuropsychiatric state and STN neurophysiology OFF medication. A.** The boxplot presents the distribution of the NSS of all subjects in the stimulation OFF (orange) and ON (red) conditions (OFF medication). **B.** Shows the power spectrum from 4-12 Hz averaged across all subjects ±SD in the stimulation OFF and ON condition when medication is OFF. No significant difference in power was found. **C.** Illustrates the Euclidean distance from the average sensing contact location using the chronic configuration to the centroid of the limbic STN in the left and right hemisphere. **D.** Presents the relationship between the NSS delta and the difference in the power spectrum between the stimulation ON and OFF condition for each frequency bin from 4-12 Hz. No significant relationship was observed. STN, subthalamic nucleus; LFP, local field potentials; DBS, deep brain stimulation; NSS, neuropsychiatric state score; SD, standard deviation.

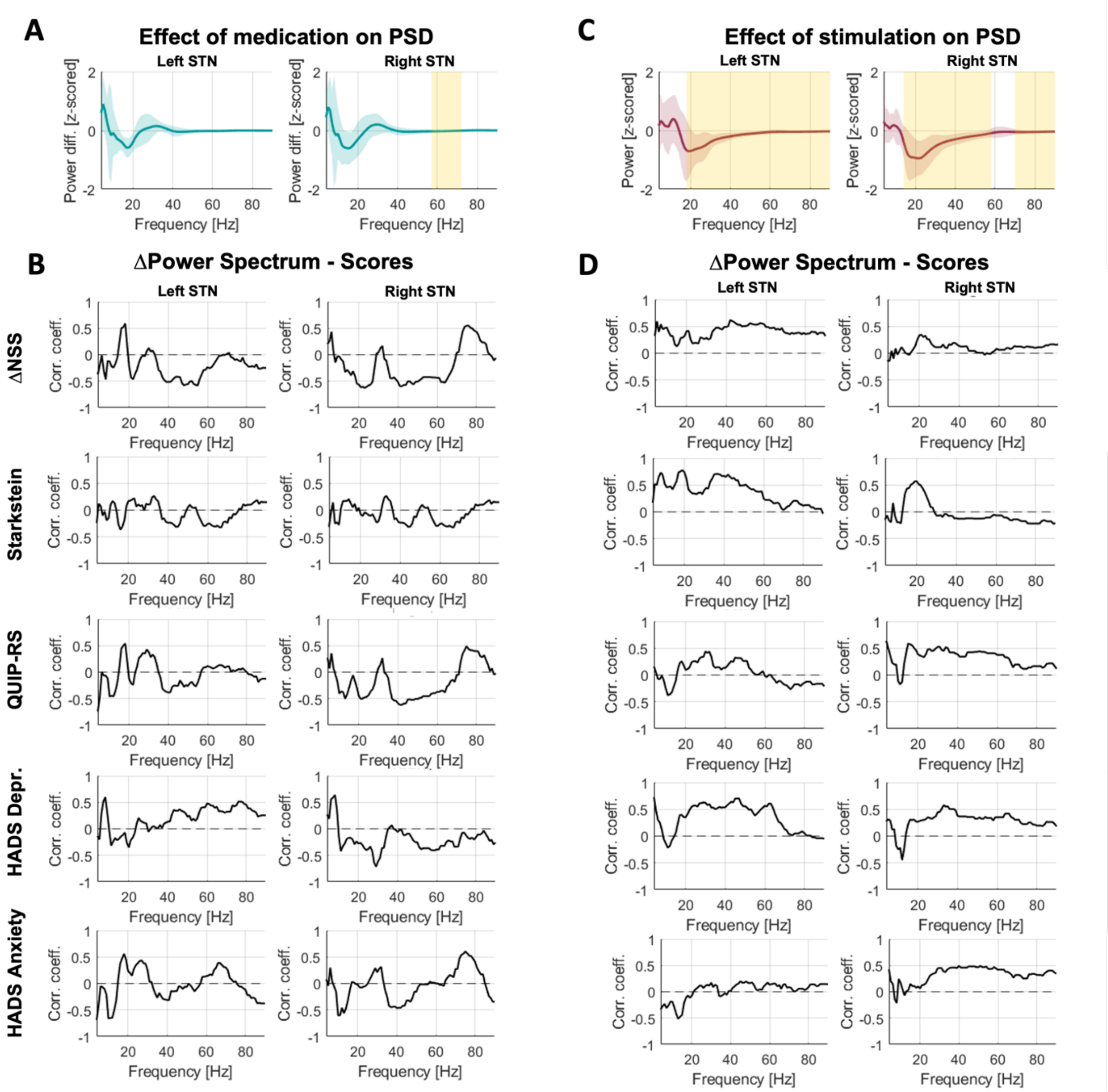

Supplementary figure 12. **Correlation of Power change and neuropsychiatric scores. A.** Shows the power difference from 4-90 Hz induced by medication averaged across all subjects ±SD. The shaded area (yellow) indicates a significant difference in power. **B.** Presents the relationship between the neuropsychiatric scores and the difference in the power spectrum between the medication ON and OFF condition for each frequency bin from 4-90 Hz. No significant relationship was observed. **C.** Shows the power difference from 4-90 Hz induced by stimulation averaged across all subjects ±SD. The shaded area (yellow) indicates a significant difference in power. **D.** Presents the relationship between the neuropsychiatric scores and the difference in the power spectrum between the stimulation ON and OFF condition for each frequency bin from 4-90 Hz. No significant relationship was observed. NSS, neuropsychiatric state score; STN, subthalamic nucleus.

NFS OFF-items	NFS ON-items
At the moment, I feel lethargic.	At the moment, I feel sure of myself.
At the moment, it would be difficult for me to plan things properly.	Currently, I feel competent at doing things.
Currently, everything seems more unpleasant to me	Currently, ideas come to me more easily than usual.
At the moment, I lack energy for everyday activities.	I would be permanently satisfied if I could feel like I do now all the time.
At the moment, I feel low.	At the moment, I feel full of energy.
Now, I feel so low that other people must notice	I feel as if something pleasant has just happened to me.
At the moment, I am unable to relax	Now, I am able to concentrate properly on something.
At the moment, I am lacking in confidence	At the moment, I have a feeling of well-being.
At the moment, I have “jelly legs, trembling”.	Currently, I feel interested in certain things.
Now, I am tired	Currently, I feel talkative, I want to communicate.

Supplementary table 2. **NFS OFF- and ON-items.** All sub-items of the NFS questionnaire, divided into OFF- and ON-items [1].

Subject	UPDRS OFF/ON	UPDRS OFF/OFF	UPDRS ON/OFF	UPDRS ON/ON	UPDRS_L OFF/ON	UPDRS_L OFF/OFF	UPDRS_L ON/OFF	UPDRS_L ON/ON	UPDRS_R OFF/ON	UPDRS_R OFF/OFF	UPDRS_R ON/OFF	UPDRS_R ON/ON
1	23	34	18	14	8	10	5	3	4	5	4	5
2	18	51	21	14	7	19	9	6	2	15	4	1
3	36	59	21	13	11	20	7	4	10	20	6	3
4	24	43	21	10	8	14	8	2	6	17	5	2
5	35	55	30	26	16	24	11	11	8	12	10	8
6	39	50	35	22	13	19	12	9	11	12	9	5
7	31	36	15	9	14	14	7	5	14	16	7	4
8	22	43	30	22	6	11	5	6	5	9	8	3
9	23	42	21	11	6	9	5	2	5	14	6	2
10	27	33	15	9	3	5	1	0	6	6	1	0
11	25	42	33	15	9	15	12	3	10	14	12	2
12	14	54	15	4	9	23	5	1	1	21	5	0
13	38	47	33	30	11	13	7	7	9	12	7	6
14	16	29	16	9	5	11	7	2	5	11	4	3

Supplementary table 3. **MDS-UPDRS III scores.** Total score and hemi-body items of the MDS-UPDRS-III (L: left, R: right) in the medication / stimulation OFF/ON conditions. UPDRS, Movement Disorders Society - Unified Parkinson’s Disease Rating Scale part 3.

Subject	NSS OFF/ON	NSS OFF/OFF	NSS ON/ON	NSS ON/OFF	SAS	HADS Anxiety	HADS Depression	QUIP-RS
1	15	8	56	46	9	2	3	2
2	31	16	42	39	13	3	1	0
3	13	2	56	57	29	9	3	30
4	6	3	56	35	9	3	3	1
5	23	11	30	29	11	7	8	9
6	42	14	55	52	NaN	NaN	NaN	NaN
7	10	2	58	53	10	6	3	3
8	33	20	45	25	15	2	8	1
9	9	0	47	35	6	8	5	4
10	13	5	43	55	16	4	4	25
11	27	20	38	42	9	0	0	1
12	29	5	52	56	28	6	11	13
13	6	1	41	53	9	5	6	3
14	41	21	56	46	13	0	9	2

Supplementary table 4. **Neuropsychiatric scores.** Neuropsychiatric state score in the medication / stimulation OFF/ON conditions and the total score of neuropsychiatric questionnaires. NSS, neuropsychiatric state score; QUIP-RS, Questionnaire for Impulsive-Compulsive Disorders in Parkinson's Disease – Rating Scale; HADS, Hospital Anxiety and Depression Scale; SAS, Starkstein Apathy Scale.

Subject	Condition	Side	1 Hz	2 Hz	3 Hz	4 Hz	5 Hz	6 Hz	7 Hz	8 Hz	9 Hz	10 Hz	11 Hz	12 Hz
1	medOFFstimOFF	Left	16.41	16.71	15.27	10.90	9.64	9.72	9.66	9.27	8.13	7.28	7.08	6.36
1	medONstimOFF	Left	19.52	13.80	13.77	12.27	11.24	10.85	10.25	9.63	8.82	8.25	7.19	6.19
2	medOFFstimOFF	Left	31.29	37.85	23.65	15.68	13.62	12.08	10.97	10.94	10.95	10.25	10.20	10.55
2	medONstimOFF	Left	16.52	19.23	14.87	20.65	27.00	17.49	14.01	12.45	12.08	12.14	11.94	11.62
3	medOFFstimOFF	Left	40.96	44.03	41.71	40.48	25.38	26.75	32.11	32.92	23.99	17.18	13.76	12.26
3	medONstimOFF	Left	40.69	41.11	34.33	39.31	28.71	25.57	26.54	24.23	18.36	14.98	12.86	11.01
4	medOFFstimOFF	Left	18.52	21.32	20.07	16.25	13.86	15.33	18.73	32.50	34.82	18.23	10.46	9.07
4	medONstimOFF	Left	19.85	20.67	22.16	17.04	14.62	14.52	12.88	11.15	9.00	7.99	7.85	7.65
5	medOFFstimOFF	Left	23.80	23.91	19.55	13.84	11.65	11.69	11.60	12.93	14.42	15.47	15.71	14.33
5	medONstimOFF	Left	29.01	27.97	18.83	13.28	13.43	13.12	13.63	14.45	12.56	10.60	9.65	9.14
6	medOFFstimOFF	Left	33.30	25.66	32.21	23.45	19.86	19.33	24.38	25.20	23.49	19.72	20.02	20.29
6	medONstimOFF	Left	32.93	33.93	32.64	43.28	48.70	31.67	24.39	25.89	23.91	21.66	21.89	21.78
7	medOFFstimOFF	Left	65.15	58.74	32.03	17.63	16.09	20.09	29.97	31.32	28.35	28.14	23.44	18.17
7	medONstimOFF	Left	156.68	74.75	37.34	23.63	24.56	26.74	29.08	27.43	30.68	26.35	17.47	12.64
8	medOFFstimOFF	Left	41.14	33.88	25.94	18.70	16.18	14.41	13.88	14.41	14.21	13.18	11.37	9.93
8	medONstimOFF	Left	32.88	33.81	23.14	27.31	19.39	14.80	14.42	17.26	15.66	12.94	10.56	9.39
9	medOFFstimOFF	Left	44.26	25.54	15.94	12.93	11.91	14.01	17.07	20.54	20.38	16.84	13.39	12.10
9	medONstimOFF	Left	31.54	22.42	14.70	13.91	13.36	17.26	26.89	35.62	25.47	15.22	10.93	9.60
10	medOFFstimOFF	Left	33.87	32.85	22.35	17.16	14.68	13.53	15.30	16.35	15.32	14.62	14.85	13.29
10	medONstimOFF	Left	27.29	27.24	21.82	17.28	15.40	13.95	14.69	14.25	12.35	12.86	13.94	12.53
11	medOFFstimOFF	Left	25.99	22.67	16.48	12.43	11.26	9.85	10.69	10.24	8.59	8.08	8.25	9.00
11	medONstimOFF	Left	32.54	27.31	19.73	14.09	12.84	10.59	11.01	10.70	9.57	9.53	9.20	8.42
12	medOFFstimOFF	Left	50.34	59.08	40.17	26.60	20.24	18.13	16.64	16.66	17.63	19.78	23.02	22.24
12	medONstimOFF	Left	49.49	56.73	36.92	25.48	21.90	32.73	21.30	20.30	20.71	19.03	18.16	15.39
13	medOFFstimOFF	Left	35.60	21.28	15.69	15.05	12.67	12.28	10.87	10.92	9.97	10.07	11.28	11.46
13	medONstimOFF	Left	55.83	42.89	26.92	15.85	13.62	11.97	11.30	10.76	10.58	11.37	10.50	9.23

Subject	Condition	Side	1 Hz	2 Hz	3 Hz	4 Hz	5 Hz	6 Hz	7 Hz	8 Hz	9 Hz	10 Hz	11 Hz	12 Hz
14	medOFFstimOFF	Left	18.97	18.05	15.15	12.65	10.53	10.30	10.86	10.88	10.46	10.56	10.62	10.71
14	medONstimOFF	Left	17.26	20.86	18.19	22.31	21.97	20.70	20.71	19.91	18.78	18.29	17.19	14.52
1	medOFFstimOFF	Right	32.20	28.44	23.44	18.54	12.91	12.21	11.02	10.54	9.73	9.65	9.15	8.17
1	medONstimOFF	Right	33.92	24.24	19.35	16.72	14.43	12.86	12.27	11.64	11.50	11.25	9.93	8.24
2	medOFFstimOFF	Right	29.22	30.84	21.02	16.84	13.70	12.04	12.03	14.84	17.62	19.15	18.16	15.77
2	medONstimOFF	Right	25.94	26.93	16.62	14.75	16.48	12.39	12.00	15.36	19.34	21.08	18.59	15.03
3	medOFFstimOFF	Right	32.55	26.77	20.43	19.80	14.99	16.85	25.75	26.95	18.34	12.47	10.11	9.44
3	medONstimOFF	Right	23.77	24.51	18.78	18.73	16.61	18.57	21.20	18.52	14.05	11.39	9.88	8.79
4	medOFFstimOFF	Right	46.02	46.69	29.99	31.87	31.64	37.62	49.03	83.86	76.42	39.05	24.65	21.22
4	medONstimOFF	Right	63.92	57.14	44.97	36.33	33.46	28.26	29.07	25.09	21.77	19.24	18.00	17.32
5	medOFFstimOFF	Right	28.34	20.44	15.57	11.82	11.10	10.96	11.26	11.55	12.58	12.61	12.92	11.92
5	medONstimOFF	Right	23.30	21.35	14.98	12.35	10.57	10.98	11.48	12.45	11.82	10.30	9.10	8.12
6	medOFFstimOFF	Right	26.09	19.28	16.95	13.56	15.51	18.70	20.44	18.91	17.40	18.62	22.38	26.50
6	medONstimOFF	Right	23.28	24.67	22.72	23.45	32.39	24.84	20.50	20.04	18.71	17.84	16.91	16.84
7	medOFFstimOFF	Right	94.79	89.86	52.75	27.31	19.62	18.50	24.31	25.49	22.36	19.58	16.32	14.11
7	medONstimOFF	Right	144.27	101.54	52.30	28.74	25.16	25.28	26.36	24.51	21.32	17.41	13.82	11.62
8	medOFFstimOFF	Right	73.49	44.45	32.14	21.57	16.59	16.62	16.50	16.84	18.29	16.98	14.55	12.53
8	medONstimOFF	Right	46.68	32.74	22.20	18.18	17.58	17.50	17.02	18.73	18.61	16.42	12.80	10.83
9	medOFFstimOFF	Right	31.42	36.37	27.57	23.65	24.71	27.83	29.44	32.74	37.09	33.59	29.15	27.34
9	medONstimOFF	Right	32.55	34.91	43.95	48.02	48.42	52.55	62.77	80.92	51.04	25.35	16.82	14.79
10	medOFFstimOFF	Right	52.87	32.50	21.02	17.51	14.96	14.93	16.34	17.56	17.46	17.04	18.36	17.24
10	medONstimOFF	Right	42.95	34.42	24.66	20.74	16.99	14.49	13.76	13.00	12.73	15.03	16.90	13.73
11	medOFFstimOFF	Right	45.42	38.22	24.08	17.49	15.92	16.92	17.99	18.45	14.84	13.02	13.57	15.07
11	medONstimOFF	Right	65.22	47.18	34.24	20.19	17.75	15.78	15.52	14.52	12.49	12.59	12.14	12.07
12	medOFFstimOFF	Right	51.96	53.00	32.51	18.58	14.66	14.82	17.60	20.61	25.05	29.54	30.80	26.78
12	medONstimOFF	Right	26.59	32.98	23.99	22.13	22.78	38.69	22.58	23.07	25.92	25.30	23.84	19.20
13	medOFFstimOFF	Right	21.81	17.75	16.57	15.03	14.76	14.91	14.70	16.06	17.79	25.95	38.31	41.87
13	medONstimOFF	Right	38.38	27.98	24.89	23.14	19.09	19.10	21.81	21.63	22.99	27.02	28.30	26.24

Subject	Condition	Side	1 Hz	2 Hz	3 Hz	4 Hz	5 Hz	6 Hz	7 Hz	8 Hz	9 Hz	10 Hz	11 Hz	12 Hz
14	medOFFstimOFF	Right	23.38	23.14	18.03	16.51	15.23	13.28	14.27	15.06	14.56	14.88	14.67	13.94
14	medONstimOFF	Right	22.45	25.53	26.30	30.82	32.97	30.68	31.16	28.98	30.28	31.60	29.16	23.80

Supplementary table 5. **Ventral low frequency power.** Spectral power of LFPs recorded with the ventral sensing configuration (0-2).

Subject	Condition	Side	1 Hz	2 Hz	3 Hz	4 Hz	5 Hz	6 Hz	7 Hz	8 Hz	9 Hz	10 Hz	11 Hz	12 Hz
1	medOFFstimON	Left	23.23	19.74	15.43	14.55	12.90	11.77	11.13	10.61	9.59	8.48	8.01	7.45
1	medOFFstimOFF	Left	21.60	21.64	17.71	14.67	13.12	12.70	12.11	10.53	9.53	8.83	8.23	7.58
1	medONstimOFF	Left	36.81	20.71	15.25	14.23	13.45	13.11	12.22	11.35	10.10	9.57	8.52	7.11
1	medONstimON	Left	44.36	26.09	17.95	15.56	13.61	12.42	11.98	11.67	10.40	9.03	8.08	7.36
2	medOFFstimON	Left	37.20	29.44	21.83	18.93	24.68	22.62	20.09	17.64	19.89	21.63	20.39	18.71
2	medOFFstimOFF	Left	47.18	29.49	31.11	30.58	21.99	16.66	12.45	12.39	11.98	11.17	10.70	11.04
2	medONstimOFF	Left	54.60	26.08	26.84	28.95	32.88	21.00	16.03	14.11	12.96	12.16	11.23	9.89
2	medONstimON	Left	43.02	28.73	19.04	18.05	24.49	25.57	21.91	17.70	18.01	20.01	20.06	18.37
3	medOFFstimON	Left	45.12	27.88	35.08	24.45	23.24	21.48	23.26	24.37	19.49	16.53	14.60	13.14
3	medOFFstimOFF	Left	45.07	28.62	41.95	37.12	24.24	20.06	19.99	16.75	12.17	10.36	9.03	8.43
3	medONstimOFF	Left	51.89	42.23	34.90	37.01	26.43	19.45	18.86	15.14	12.15	10.46	9.07	8.35
3	medONstimON	Left	111.65	67.10	32.79	25.12	20.28	21.96	24.03	22.74	17.02	14.98	13.65	12.51
4	medOFFstimON	Left	20.04	20.39	19.75	16.58	12.93	12.04	12.20	12.80	12.30	11.29	10.88	9.78
4	medOFFstimOFF	Left	21.59	20.83	22.35	17.72	15.58	15.48	15.14	19.52	22.93	16.23	12.49	11.32
4	medONstimOFF	Left	20.23	19.59	21.64	19.48	17.36	13.89	13.00	11.10	9.98	9.57	8.70	7.81
4	medONstimON	Left	22.89	18.88	19.02	14.74	13.41	11.78	11.84	11.63	11.08	10.83	10.55	9.71
5	medOFFstimON	Left	36.48	23.23	26.97	26.06	18.72	18.33	18.19	19.23	17.49	14.89	13.85	13.01
5	medOFFstimOFF	Left	34.30	30.20	21.86	18.05	16.58	16.61	17.42	18.76	17.30	14.62	12.33	10.81
5	medONstimOFF	Left	37.17	30.02	24.12	19.38	18.56	18.05	16.96	16.45	14.48	12.17	11.09	10.13
5	medONstimON	Left	34.77	27.98	25.72	27.86	21.15	21.16	21.56	21.60	19.38	17.12	14.35	12.58
6	medOFFstimON	Left	15.52	18.72	35.71	16.22	12.77	12.53	12.37	13.23	13.87	14.51	15.86	17.86
6	medOFFstimOFF	Left	21.14	15.04	17.47	11.79	11.36	11.91	13.14	13.46	13.45	13.79	15.82	18.56
6	medONstimOFF	Left	22.33	20.26	17.20	18.97	21.64	18.13	14.24	13.97	14.01	13.29	12.89	13.03
6	medONstimON	Left	22.00	21.85	23.05	23.93	23.14	18.18	16.70	17.16	14.81	13.13	12.44	11.87
7	medOFFstimON	Left	44.50	42.76	24.84	14.20	12.10	13.17	15.96	19.31	25.29	30.18	25.33	17.42
7	medOFFstimOFF	Left	60.55	60.42	30.85	17.60	13.46	16.82	22.80	23.52	22.66	22.58	18.39	14.85
7	medONstimOFF	Left	55.98	51.71	28.98	20.71	23.14	22.07	20.49	21.15	19.07	16.28	12.55	10.18
7	medONstimON	Left	69.65	34.03	24.73	15.85	17.72	25.29	28.09	25.54	24.74	22.85	16.50	11.96

Subject	Condition	Side	1 Hz	2 Hz	3 Hz	4 Hz	5 Hz	6 Hz	7 Hz	8 Hz	9 Hz	10 Hz	11 Hz	12 Hz
8	medOFFstimON	Left	89.22	37.71	27.12	30.66	34.61	24.16	21.62	26.16	23.36	23.52	25.19	25.77
8	medOFFstimOFF	Left	102.33	36.41	30.17	24.49	14.44	12.37	12.19	12.88	13.43	13.33	12.02	11.29
8	medONstimOFF	Left	73.08	30.55	19.31	17.61	14.18	12.97	12.12	12.74	14.06	12.98	11.68	11.13
8	medONstimON	Left	44.77	29.78	19.08	18.99	19.40	16.00	16.00	15.63	15.92	14.81	12.95	11.22
9	medOFFstimON	Left	26.77	19.74	16.12	16.14	15.38	19.02	18.40	20.05	17.78	15.69	14.93	13.54
9	medOFFstimOFF	Left	19.99	22.48	18.02	16.89	15.60	18.33	21.92	26.47	24.82	21.77	19.90	18.83
9	medONstimOFF	Left	27.49	25.03	21.60	23.46	21.76	28.48	37.72	47.52	33.31	17.85	12.01	10.14
9	medONstimON	Left	77.73	23.74	22.53	19.12	23.86	29.03	38.38	41.26	27.31	18.62	14.93	12.65
10	medOFFstimON	Left	27.38	20.07	20.18	18.43	14.11	12.85	14.21	14.32	13.57	12.76	12.64	11.71
10	medOFFstimOFF	Left	14.25	15.37	14.08	11.90	10.68	11.34	12.31	13.24	13.02	13.44	13.52	11.23
10	medONstimOFF	Left	25.86	21.12	18.36	14.20	12.97	12.26	12.30	11.09	9.89	9.65	9.98	9.21
10	medONstimON	Left	34.88	24.64	17.22	15.27	14.45	13.19	14.19	14.13	12.64	11.20	11.29	10.36
11	medOFFstimON	Left	34.37	22.06	16.16	13.30	12.64	11.65	10.71	11.05	10.14	10.04	10.73	12.19
11	medOFFstimOFF	Left	21.50	20.22	16.49	13.51	11.65	11.11	10.62	10.45	9.08	7.88	7.55	7.40
11	medONstimOFF	Left	22.39	26.38	19.96	15.56	13.04	10.89	10.57	9.36	7.91	7.43	7.19	6.92
11	medONstimON	Left	31.05	24.88	18.62	13.63	12.90	12.35	12.79	10.82	9.74	9.27	9.12	9.04
12	medOFFstimON	Left	57.95	55.15	41.83	30.01	23.53	20.96	20.92	19.92	21.18	21.57	22.08	19.38
12	medOFFstimOFF	Left	51.09	62.24	43.68	28.58	21.10	21.07	20.76	19.69	20.41	20.83	21.80	19.02
12	medONstimOFF	Left	43.19	48.74	39.05	26.63	20.56	23.66	20.38	22.36	23.41	21.26	19.17	15.94
12	medONstimON	Left	55.78	51.44	42.25	30.36	23.26	22.84	20.06	20.59	22.84	21.57	18.38	14.90
13	medOFFstimON	Left	16.42	17.14	13.68	12.44	10.72	10.97	9.08	8.88	8.26	8.62	9.00	8.43
13	medOFFstimOFF	Left	16.31	17.85	15.54	12.11	12.06	10.30	10.07	9.64	9.48	9.87	10.76	11.77
13	medONstimOFF	Left	42.26	25.12	20.78	14.77	14.96	12.69	12.41	12.01	12.69	14.87	15.14	14.43
13	medONstimON	Left	34.73	22.88	17.42	14.01	10.92	10.13	9.92	9.65	8.80	8.60	8.95	8.32
14	medOFFstimON	Left	28.28	57.40	33.42	27.06	16.90	21.28	22.12	16.17	13.44	12.79	12.37	11.88
14	medOFFstimOFF	Left	38.66	54.04	39.84	17.38	14.39	15.68	14.50	12.10	10.26	9.81	8.98	8.10
14	medONstimOFF	Left	23.68	27.11	29.00	22.76	16.62	14.61	15.08	13.86	11.62	10.78	9.64	8.30
14	medONstimON	Left	27.51	44.66	22.83	24.24	17.91	19.55	19.01	15.75	14.58	13.74	13.67	13.01

Subject	Condition	Side	1 Hz	2 Hz	3 Hz	4 Hz	5 Hz	6 Hz	7 Hz	8 Hz	9 Hz	10 Hz	11 Hz	12 Hz
1	medOFFstimON	Right	27.09	27.52	23.71	22.69	23.41	20.13	17.70	16.35	15.94	14.09	12.54	11.57
1	medOFFstimOFF	Right	27.69	26.56	25.46	22.86	18.17	17.96	14.96	13.84	13.15	12.50	11.49	10.45
1	medONstimOFF	Right	42.07	30.27	24.76	22.87	18.99	17.63	16.21	14.85	14.19	13.34	12.56	11.20
1	medONstimON	Right	28.19	32.26	30.41	26.63	20.86	22.19	21.41	19.65	19.40	17.51	15.97	14.43
2	medOFFstimON	Right	24.97	18.78	13.06	12.59	10.85	11.15	10.93	10.10	10.53	10.69	9.71	9.06
2	medOFFstimOFF	Right	28.51	30.21	20.05	17.52	12.57	10.95	10.56	10.22	10.45	10.65	10.10	10.33
2	medONstimOFF	Right	16.39	18.35	15.84	17.83	19.01	13.16	11.80	10.99	10.14	10.50	10.00	9.14
2	medONstimON	Right	22.72	16.98	12.38	10.72	11.37	10.83	11.89	9.85	9.92	9.60	8.78	8.10
3	medOFFstimON	Right	76.52	32.62	33.77	28.16	20.57	17.58	21.13	23.21	18.49	14.95	13.09	12.09
3	medOFFstimOFF	Right	110.70	37.41	29.90	25.67	19.06	18.00	21.10	20.39	14.52	11.45	10.20	9.60
3	medONstimOFF	Right	98.50	42.83	30.76	27.28	20.84	19.19	19.54	15.42	12.48	11.45	10.55	9.46
3	medONstimON	Right	104.90	56.74	27.79	22.47	19.63	21.79	23.15	19.32	15.62	14.36	13.28	12.21
4	medOFFstimON	Right	31.28	24.07	20.01	22.43	15.24	14.66	15.53	18.81	18.64	15.88	14.48	12.90
4	medOFFstimOFF	Right	27.04	26.13	26.29	20.51	20.08	19.70	19.79	21.04	20.75	17.65	15.57	14.50
4	medONstimOFF	Right	28.19	28.94	28.97	23.69	22.72	16.83	15.42	13.67	13.62	12.52	11.03	10.21
4	medONstimON	Right	27.41	22.85	20.91	19.92	15.06	14.16	14.86	14.45	13.61	12.62	12.21	11.31
5	medOFFstimON	Right	34.97	19.62	21.68	21.85	16.43	17.21	16.79	16.94	15.57	14.14	13.46	13.36
5	medOFFstimOFF	Right	27.96	22.24	14.65	13.07	12.63	13.70	15.40	15.18	14.23	13.56	13.47	13.17
5	medONstimOFF	Right	26.63	22.48	19.57	15.43	13.04	13.38	13.49	12.39	12.24	11.48	10.64	10.16
5	medONstimON	Right	21.02	20.84	22.39	23.59	15.52	14.55	14.49	13.83	13.13	12.15	11.05	10.37
6	medOFFstimON	Right	35.51	27.94	43.19	19.61	16.64	15.09	16.00	16.36	16.89	17.00	19.40	23.17
6	medOFFstimOFF	Right	38.50	20.48	21.03	16.13	15.80	15.55	17.42	16.47	15.94	15.86	16.47	18.82
6	medONstimOFF	Right	34.78	25.72	21.59	19.21	17.86	16.82	16.56	15.77	14.79	13.86	12.66	11.85
6	medONstimON	Right	37.49	25.70	31.73	30.87	24.43	20.43	17.39	18.21	16.50	14.45	13.27	12.26
7	medOFFstimON	Right	64.05	76.65	42.27	22.85	14.99	15.20	19.25	21.05	24.13	27.85	23.82	16.86
7	medOFFstimOFF	Right	57.28	70.85	42.59	24.93	17.63	18.33	24.93	25.83	22.01	19.48	16.39	13.85
7	medONstimOFF	Right	89.77	70.99	39.76	24.01	23.33	25.33	24.08	22.46	19.73	16.13	12.54	10.34
7	medONstimON	Right	62.29	54.78	32.52	19.84	22.11	32.22	36.34	31.24	26.56	23.43	16.68	11.74

Subject	Condition	Side	1 Hz	2 Hz	3 Hz	4 Hz	5 Hz	6 Hz	7 Hz	8 Hz	9 Hz	10 Hz	11 Hz	12 Hz
8	medOFFstimON	Right	77.97	35.13	23.69	17.22	17.60	15.36	16.56	22.15	23.77	24.35	19.46	13.87
8	medOFFstimOFF	Right	79.70	42.02	29.93	20.53	17.38	17.86	18.34	19.87	21.12	19.71	15.98	12.87
8	medONstimOFF	Right	48.16	34.51	21.68	19.73	17.87	20.19	19.06	21.98	26.00	21.48	14.65	11.90
8	medONstimON	Right	75.60	36.06	18.74	16.90	18.26	17.65	17.53	22.33	23.77	20.28	14.62	11.70
9	medOFFstimON	Right	39.61	34.04	27.48	27.40	25.95	33.49	27.01	27.25	25.54	23.24	21.28	19.63
9	medOFFstimOFF	Right	30.11	24.03	25.00	21.69	23.75	23.70	23.02	23.46	20.19	17.42	17.03	17.81
9	medONstimOFF	Right	30.39	29.25	24.75	22.64	25.74	27.82	38.08	51.10	40.12	24.31	17.37	15.25
9	medONstimON	Right	89.55	77.18	36.52	29.66	33.74	34.46	35.89	40.27	32.15	26.34	22.63	20.05
10	medOFFstimON	Right	43.07	28.56	27.76	27.65	20.76	22.64	24.98	26.22	26.04	26.95	25.52	21.38
10	medOFFstimOFF	Right	37.81	28.05	21.34	15.69	14.25	15.40	18.85	21.73	25.14	30.67	32.60	26.11
10	medONstimOFF	Right	42.38	29.69	23.86	23.18	21.65	18.35	16.99	15.62	14.13	16.80	20.30	18.13
10	medONstimON	Right	38.89	27.58	22.15	22.57	20.63	19.35	18.79	17.22	15.61	15.37	17.03	15.32
11	medOFFstimON	Right	32.12	19.25	15.29	12.75	10.71	10.87	11.63	11.38	10.43	9.60	11.34	13.65
11	medOFFstimOFF	Right	25.28	21.19	16.30	12.95	11.88	11.18	10.81	10.86	9.77	9.38	9.38	10.32
11	medONstimOFF	Right	23.24	24.93	19.43	16.13	13.66	11.53	10.69	10.33	8.96	8.32	8.08	7.85
11	medONstimON	Right	26.85	20.32	17.29	14.98	11.83	11.83	12.22	11.65	9.54	9.10	9.75	10.36
12	medOFFstimON	Right	53.26	47.39	31.14	19.78	16.90	16.88	20.01	23.29	26.05	26.66	28.84	24.66
12	medOFFstimOFF	Right	65.26	55.99	31.67	20.27	17.48	16.70	19.96	25.82	31.00	33.46	34.74	28.67
12	medONstimOFF	Right	36.90	36.65	25.95	23.36	21.83	25.00	23.68	25.94	28.73	27.75	26.60	21.50
12	medONstimON	Right	35.97	32.57	22.08	18.86	15.94	16.30	19.37	20.95	21.87	21.24	21.73	17.83
13	medOFFstimON	Right	28.82	25.36	19.52	20.04	19.96	14.70	12.96	11.72	11.34	11.80	12.22	11.74
13	medOFFstimOFF	Right	25.28	26.62	22.56	16.73	14.79	13.88	12.43	12.86	11.94	13.53	15.60	16.80
13	medONstimOFF	Right	51.33	42.07	27.77	22.40	17.72	17.86	18.10	17.38	17.37	18.51	20.00	20.53
13	medONstimON	Right	47.80	36.36	22.09	21.24	18.10	16.72	15.03	13.96	12.42	11.74	11.40	11.54
14	medOFFstimON	Right	20.15	26.53	22.98	19.54	15.76	16.14	16.39	16.44	14.33	13.06	12.63	11.87
14	medOFFstimOFF	Right	25.28	34.82	25.23	17.52	15.85	14.69	14.71	13.51	13.72	13.11	11.94	11.57
14	medONstimOFF	Right	25.88	30.53	26.73	23.21	20.98	20.27	18.61	18.16	17.80	17.76	18.27	17.61
14	medONstimON	Right	24.38	35.68	32.49	21.14	16.22	17.21	18.10	17.62	15.98	15.13	14.96	14.78

Supplementary table 6. **Low frequency power measured with chronic configuration.** Spectral power of LFPs recorded with the sensing contacts surrounding the contacts used for chronic stimulation.

References

1. Schmitt, E. *et al.* The Neuropsychiatric Fluctuations Scale for Parkinson's Disease: A Pilot Study. *Mov Disord Clin Pract* 5, 265–272 (2018).
